# Neural network potentials for accelerated metadynamics of oxygen reduction kinetics at Au–water interfaces†

**DOI:** 10.1039/d2sc06696c

**Published:** 2023-03-13

**Authors:** Xin Yang, Arghya Bhowmik, Tejs Vegge, Heine Anton Hansen

**Affiliations:** a Department of Energy Conversion and Storage, Technical University of Denmark Anker Engelunds Vej, 2800 Kgs Lyngby Denmark heih@dtu.dk

## Abstract

The application of *ab initio* molecular dynamics (AIMD) for the explicit modeling of reactions at solid–liquid interfaces in electrochemical energy conversion systems like batteries and fuel cells can provide new understandings towards reaction mechanisms. However, its prohibitive computational cost severely restricts the time- and length-scales of AIMD. Equivariant graph neural network (GNN) based accurate surrogate potentials can accelerate the speed of performing molecular dynamics after learning on representative structures in a data efficient manner. In this study, we combined uncertainty-aware GNN potentials and enhanced sampling to investigate the reactive process of the oxygen reduction reaction (ORR) at an Au(100)–water interface. By using a well-established active learning framework based on CUR matrix decomposition, we can evenly sample equilibrium structures from MD simulations and non-equilibrium reaction intermediates that are rarely visited during the reaction. The trained GNNs have shown exceptional performance in terms of force prediction accuracy, the ability to reproduce structural properties, and low uncertainties when performing MD and metadynamics simulations. Furthermore, the collective variables employed in this work enabled the automatic search of reaction pathways and provide a detailed understanding towards the ORR reaction mechanism on Au(100). Our simulations identified the associative reaction mechanism without the presence of *O and a low reaction barrier of 0.3 eV, which is in agreement with experimental findings. The methodology employed in this study can pave the way for modeling complex chemical reactions at electrochemical interfaces with an explicit solvent under ambient conditions.

## Abstract 2

The combination of metadynamics and equivariant graph neural network potentials enables the full simulation of oxygen reduction at Au(100)–water interfaces. This strategy can be used for modeling multiple chemical reactions.

## Graphical abstract

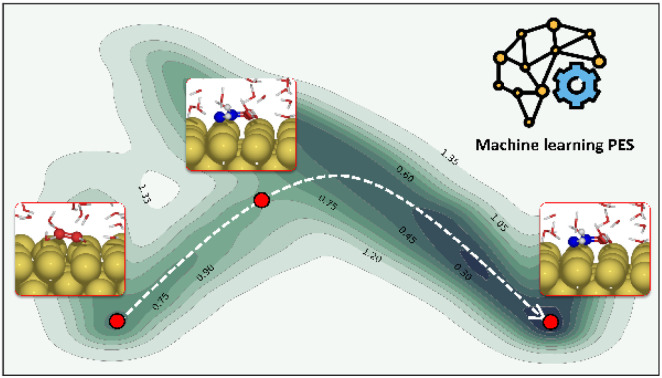

## 1 Introduction

Over the past several decades, density functional theory (DFT) calculations have been extensively used for developing novel electrocatalysts towards the oxygen reduction reaction (ORR) by taking advantage of well-developed theoretical methods^1–5^ (*e.g.*, free energy diagrams, volcano plots, and d-band theory) for predicting catalytic activities. Nevertheless, most of these calculations oversimplify the operating conditions of catalysts by either modelling liquid water at the electrolyte–electrode interface as static water layers,^6–9^ implicitly representing them *via* dielectric continuum models,^10–12^ or even absolutely ignoring the effect of solvents.^13–16^ These limitations may lead to erroneous evaluation of activity trends of catalysts as compared to experiments, for example, the oxygen reduction reaction on gold in alkaline electrolytes.^17,18^ Including solvent molecules for electrolyte–electrode interface simulations and investigating their dynamical effects could offer us a better understanding towards the reaction mechanisms of the ORR and may resolve the conflicts between theoretical calculations and experiments.

While *ab initio* molecular dynamics (AIMD) is capable of capturing the dynamics of liquid water, it is prohibitively expensive for large length-scale and long time-scale simulations. For instance, the time-averaged metrics (*e.g.*, energy and temperature) of AIMD simulations can differ significantly if started from different initial configurations, while these discrepancies could be greatly mitigated if the model system is equilibrated and sampled from long enough trajectories.^19–21^ The prohibitive computational cost severely limits the equilibration and sampling time scales of AIMD to only a few ps, which may significantly impair the reliability of such studies.^19,22–28^

Recently, advances in machine learning have played great roles in aiding the design and discovery of transition metal based catalysts.^29,30^ By learning from data, machine learning tools can make fast predictions to find target catalysts and provide valuable insights into the nature of the reaction, which enable high-throughput screening of catalysts from a broad chemical space and automated catalyst design.^31–33^ In particular, neural network potentials (NNPs) have shown great promise at fitting the potential energy surface (PES) of reactive model systems by training on reference configurations that well describe the representative atomic environments.^34–37^ This approach could speed up MD simulations by several orders of magnitude whilst retaining the accuracy comparable to AIMD, which enables us to considerably extend the time scale and length scale of MD simulations without compromising accuracy. Initially proposed architectures of neural network potentials learned the force field by leveraging handcrafted features based on distance and angle information to capture the characteristics of local atomic environments.^38–40^ Behler–Parrinello neural network potential is the first example in which the Cartesian coordinates of atoms are transformed to rotational and translational invariant atomic-centered symmetry functions.^38,39^ Recent advances in graph neural networks (GNNs) for molecule graphs have made it possible to learn representative features from the atomic structure *via* a graph message-passing scheme.^41–45^ State-of-the-art GNN models leverage the rotation equivariant representation of node features (*i.e.*, features of atomic environments) to provide more accurate force predictions, which can be essential in MD simulations.^44–46^ In spite of numerous novel machine learning methods for fitting PES and MD simulations driven by NNPs,^20,21,47–50^ there are few studies on simulating nonequilibrium dynamics and reactions. We have yet to find out any study performing sampling of rare events that govern chemical reactions with NNPs.^28,51,52^ Taking the ORR as an example, although NNPs can significantly accelerate MD simulations, the time scale of reactive simulation of the ORR is still inaccessible, not to mention the complex ambient conditions of the catalysts. Due to the rapid development of enhanced sampling techniques like metadynamics^53,54^ (MetaD), high accuracy sampling of PES has been possible for such rare events. We envision that combining enhanced sampling methods together with high-fidelity NNPs can enable full simulation of slow chemical reactions on an atomic scale within affordable computational cost.

In this paper, we present the full atomic simulation of the ORR at an Au(100)–water interface done using metadynamics simulations accelerated by equivariant graph neural network potentials.^43^ The gold electrode has been extensively studied as an ORR electrocatalyst, while its exceptional activity, especially in alkaline media, is still not well-explained.^17,18,55,56^ This case could well demonstrate the power of our proposed simulation paradigm towards modeling of rare chemical reactions at solid–liquid interfaces. Compared to non-reactive MD performed with NNPs, a major challenge of simulating rare events like the ORR is to ensure that the machine learning model encompasses a vast configurational space far away from equilibrium. This requires adaptive sampling of representative reference structures from MD and MetaD simulations, particularly transition states that are rarely visited. In addition, quantitatively evaluating the reliability of NNPs for describing the PES in the configurational space of interest is also indispensable. Here we adopt an active learning approach based on CUR matrix decomposition^57,58^ to sample representative reference structures from MD and MetaD simulations. This method enables us to representatively sample the vast configurational spaces of the ORR at the solid–liquid interface with minimal human intervention and significantly reduced computational cost. Our MD and MetaD simulations are uncertainty aware, demonstrating robust and reliable modeling of full atomic simulation of the ORR with NNPs.

## 2 Computational details

### 2.1 Active learning framework

**Fig. 1:**
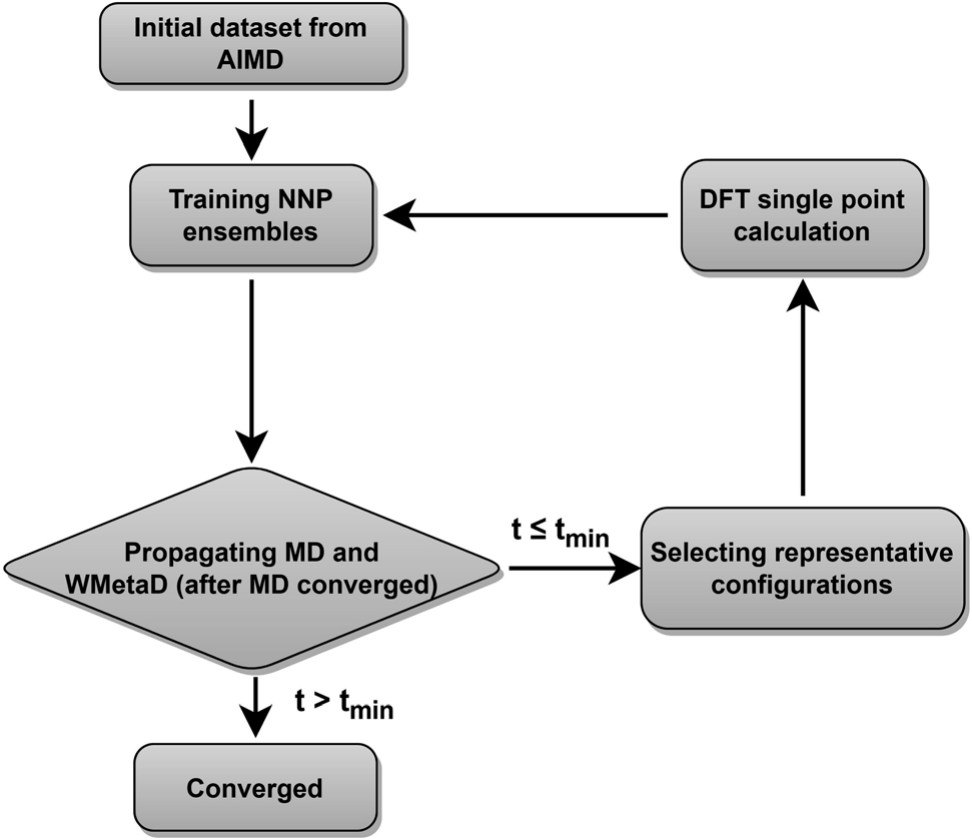
Active learning procedures used to train the neural network potentials.

Our neural network potentials are constructed based on an active learning framework utilizing CUR decomposition based selective sampling as demonstrated in Fig. 1. First, an initial dataset was generated by selectively sampling reference structures from several AIMD trajectories of Au(100)–water interfaces. Multiple interface structures with different numbers of hydroxyl or oxygen molecules are considered to ensure the diversity and versatility of the training dataset and to further study the impact of adsorbates on the dynamics of solvents. The initial AIMD trajectories contain several hundreds of thousands of configurations. Using all of them would make the training of NNPs very slow. Many structures are similar, and thus models do not capture new correlations when all of those are used simultaneously. Therefore, it is crucial to sample only those structures that are representative and informative from these trajectories. We first select structures from AIMD trajectories one in every 50 MD steps, reducing the number of candidate configurations to several tens of thousands. Then the CUR matrix decomposition method^57,58^ is employed to further refine the training dataset without losing too much information. Given an N × M data matrix X with its rows corresponding to *N* atoms and its columns corresponding to *M* fingerprints, the objective of CUR is to minimize the information loss after ruling out some rows and columns, while minimizing the number of rows and columns to be selected. We also add an extra term in the objective function to maximize the Euclidean distance between different atomic environments to ensure the diversity of sampled structures. In this process, the importance of each row and column in the data matrix can be evaluated, and the representative configurations and fingerprints can be jointly sampled. Here the fingerprints of atoms in candidate structures are described by Behler–Parrinello symmetry functions,^38,39^ which have been extensively used for fitting the PES for solid–liquid interface systems.^20,21,47–50^ CUR matrix decomposition also provides an efficient way for automatically selecting symmetry function parameters that are typically non-trivial.

An ensemble of neural network potentials (NNPs) was then trained on the initial dataset with 5000 reference structures after CUR selection. In order to extend their capability of exploring larger configurational space, the trained NNPs are updated adaptively in the following steps: (i) propagating MD trajectories with the trained NNP ensemble as an energy/force calculator; (ii) selecting representative reference structures from MD trajectories by using CUR decomposition; (iii) calculating these selected new data points with DFT; (iv) retraining NNPs with the expanded training dataset. Instead of propagating MD using only one NNP, we choose to combine all the trained NNPs together for the prediction of energy and forces. This strategy not only improves the predictive accuracy of our model but also provides a practical way to quantify if the model is still confident enough in the configurational space of interest. The quantification is achieved by evaluating the energy uncertainty and force uncertainty for every step *via* calculating the variance of NNPs during the MD simulation. Fig. S1† compares the calculated uncertainty and true prediction error, indicating that uncertainty is an excellent indicator for true model error. Based on the query-by-committee method, which has been widely used in active learning,^46,59,60^ the configurations with relatively large uncertainty are collected to reduce the number of candidate configurations. Subsequently, the obtained structures are further sub-sampled by CUR decomposition for DFT evaluation. By adding these carefully chosen new data in the training set, we constantly improve the model prediction for new configurational space visited by MD simulations. Combining this strategy and CUR matrix decomposition significantly reduces the number of candidate structures and ensure the diversity of structures in a sampled batch. The simulations are stopped if the uncertainties are too large or too many structures with large uncertainties are collected. The iterative training of the NNP ensemble stops once all the MD simulations can be propagated to more than *t*_min_ steps, where *t*_min_ is selected as 5 ns to ensure that the model systems are properly equilibrated and all the dynamical events are fully captured.^21,47,48^ To further investigate the ORR kinetics at the gold–water interface, the iterative training procedures are repeated in the case of MetaD simulations. Notably, as the transition states are rarely visited during MetaD runs, it is critical to include enough such configurations into our training dataset and validate our MetaD simulations *via* uncertainty quantification.

### 2.2 CUR matrix decomposition

Reference structures are adaptively sampled by CUR matrix decomposition^57,58^ from MD simulations driven by the NNPs. CUR matrix decomposition is a low rank approximation to the input matrix, indicating that the information of the matrix can be maintained after discarding some columns and rows. Given an *n* × *m* data matrix *X*, our objective is to select the least number of rows and columns from *X* to construct a subset matrix 
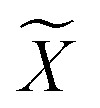
 while minimizing the information loss. To address this issue, Li *et al.* proposed the ALFS algorithm^58^ to minimize the following objective function by using an augmented Lagrange multiplier:1
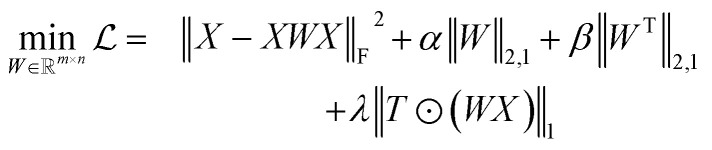
where 
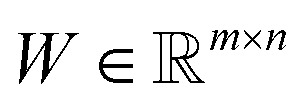
 is an auxiliary matrix that determines which rows and columns should be selected. Minimizing the *l*_2_ norm of its rows and columns corresponds to minimizing the number of selected columns and rows, respectively. The weight matrix *T* that encodes the Euclidean distance between different rows is used to maximizing the distance between selected rows, which could effectively increase the diversity of datapoints selected in an active learning batch. The regularization parameters *α*, *β*, and *γ* are used to determine the priority to minimize the row numbers, column numbers or row distance, respectively. The *l*_2_ norm of rows and columns of optimized *W* will be regarded as the importance score of each column and row in the data matrix, and the importance score of a configuration will be calculated as the average of the importance score of atoms inside it.

One thing that should be noted is that the overall data matrix obtained from an MD run can typically contain a few million to several billion entries with a feature size of more than one hundred, on which the implementation of CUR decomposition can be intractable. Instead of using the data matrix as a whole, we will split the large data matrix into smaller ones by rows, and then assess the importance of every row and column *via* CUR decomposition of the smaller matrices on-the-fly. This strategy significantly improves the efficiency and computational cost of CUR selection while has minor impact on the performance of CUR. The size of divided matrices is selected as 1000 entries and 90 features for each element generated by Behler–Parrinello symmetry functions. The parameters of symmetry function are also selected by CUR decomposition from a pool of 3000 symmetry functions.

### 2.3 AIMD and DFT single point calculations

The Au(100)–water interface is modelled as 30 H_2_O molecules on top of a (3 × 3) tetragonal Au(100) surface with four atomic layers, which will be denoted as Au(100)–30H_2_O hereafter. A vacuum layer larger than 15 Å is perpendicularly added into the model to eliminate the spurious interaction between periodic images. In order to simulate the interface with ORR intermediates, we also consider structures with one and two hydroxyls by removing the hydrogen atoms from water molecules near the slab, and a structure with one oxygen molecule on top of an Au(100) slab. These structures are denoted as Au(100)–1OH/29H_2_O, Au(100)–2OH/28H_2_O, and Au(100)–1O_2_/30H_2_O, respectively. Constant temperature MD simulations are then performed in VASP^61–64^ by using these initial configurations with a timestep of 0.5 fs and the temperature is kept at around 350 K with a Nosé–Hoover thermostat.^65^ The bottom two layers are kept fixed during the MD run for all model systems. 50 ps, 15 ps, 15 ps, and 15 ps MD simulations are conducted for Au(100)–30H_2_O, Au(100)–1OH/29H_2_O, Au(100)–2OH/28H_2_O, and Au(100)–1O_2_/30H_2_O, respectively. The reason for running shorter MD simulations on the model systems with adsorbates is that their most local structures are similar to the Au(100)–30H_2_O system. Density functional calculations are used to calculate the potential energy and the forces for propagating AIMD and labeling representative configurations sampled by active learning. We employ an energy cutoff of 350 eV for plane-wave basis expansion and a 2 × 2 × 1 Monkhorst–Pack k-grid for Brillouin zone sampling.^66^ The exchange-correlation effects are approximated by using the PBE functional combined with D3 van der Waals correction.^67,68^

### 2.4 Production molecular dynamics simulations

The production MD simulations driven by the NNP ensemble have been performed using the MD engine of the Atomic Simulation Environment (ASE) python library.^69^ The simulation box in AIMD is too small to accommodate more adsorbates and to simulate the full reaction. Furthermore, previous studies also demonstrated that notable noise in the structural properties of the model systems could be observed when using small cell sizes.^47,70^ Considering both effects and the increased computational cost for MD and labelling, we constructed a larger model with 59 H_2_O molecules on top of a (4 × 4) tetragonal Au(100) surface with four atomic layers, on which more adsorbates can be accommodated. With the presence of one to six *OH, the corresponding hydrogen atoms are removed at the interface, producing interface structures that could be denoted as Au(100)–1OH/58H_2_O, Au(100)–2OH/57H_2_O, Au(100)-3OH/56H_2_O, Au(100)–4OH/55H_2_O, Au(100)–5OH/54H_2_O, and Au(100)–6OH/53H_2_O, respectively. In order to investigate the kinetics of the ORR, the initial state structure Au(100)–1O_2_/57H_2_O is also built by removing two H_2_O molecules and placing a O_2_ molecule on top of Au(100). The momentum of model systems is initiated by a Maxwell–Boltzmann distribution with the temperature set to 350 K. The MD simulations are propagated for 5 ns by Langevin dynamics with a target temperature of 350 K, a timestep of 0.25 fs, and a friction coefficient of 0.02. It is noteworthy that a smaller time step is selected for production as it can help the MD simulations reach a longer time scale with smaller uncertainty. The uncertainties of frames in MD simulations are quantified as the variance and standard deviation (SD) of model outputs:2
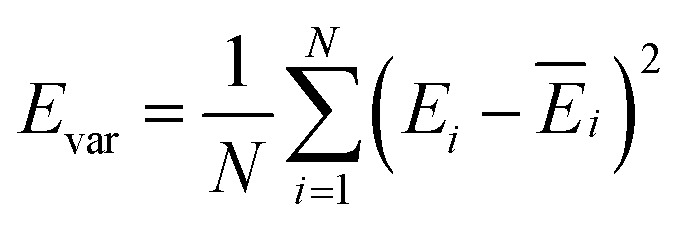
3
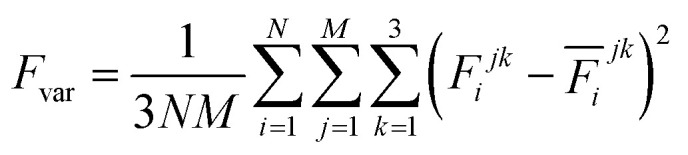
4
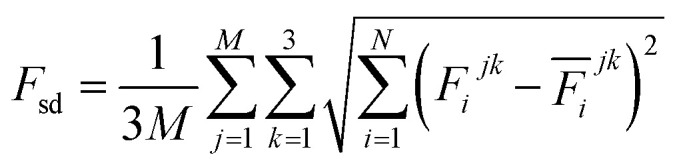
where *N* is the number of models in the ensemble, *M* is the number of atoms in a frame, and *E&x0304;* and *F&x0304;* are the average predicted energy and force, respectively. In order to ensure the reliability of MD results, the simulations will stop if *F*_sd_ is larger than 0.5 eV Å^−1^ or more than 2000 structures with *F*_sd_ larger than 0.05 ev Å^−1^ are collected.

Following the method in ref. ^19^, we calculated the formation energy of *OH as the internal energy of the Au(100)–*n*_OH_OH/(59-*n*_OH_)H_2_O interface structure, plus the internal energy of gas phase *n*_OH_/2H_2_ molecules, minus the internal energy of the Au(100)–59H_2_O interface structure.5
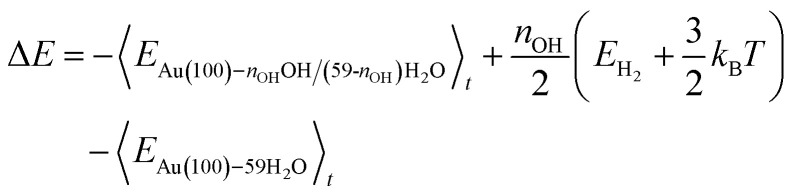


The internal energy of interface structures is calculated as the time averaged potential energy plus kinetic energy. And the internal energy of H_2_ gas molecules is calculated as the potential energy plus 3/2*k*_B_*T* because their center-of-mass motions are not included in the MD simulations. Likewise, the adsorption energy of O_2_ is calculated as follows.6
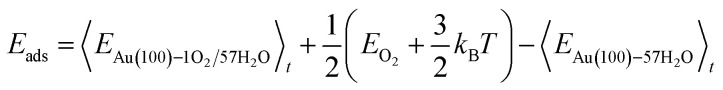


### 2.5 Metadynamics simulations

In this study, all the enhanced sampling simulations are performed with a well-tempered version of metadynamics.^71^ The production metadynamics simulations are propagated by Langevin dynamics for 2.5 ns in ASE. The calculation of collective variables and bias potential of metadynamics is achieved by using PLUMED^72–74^ which is interfaced to the ASE library. To construct the path CVs as described in the main text, the Au(100)–1O_2_/57H_2_O and Au(100)–4OH/55H_2_O interface structures are selected as two reference structures. And the coordination numbers (C_O_2__–_O_) and (C_O_2__–_H_) are used to define the configurational space of the path. The corresponding equations and parameters for calculating (C_O_2__–_O_) and (C_O_2__–_H_) are shown in Table S1†

With the defined path, the progress along the path *s* and the distance from the path *z* can be computed as:7
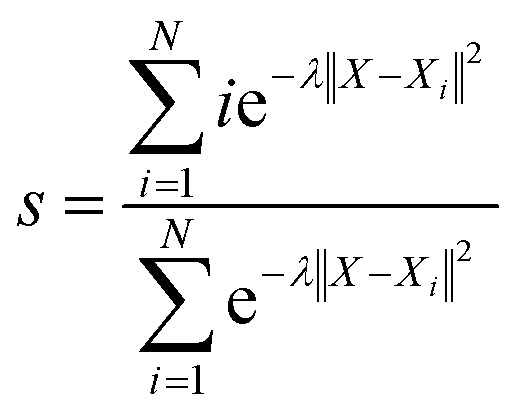
8
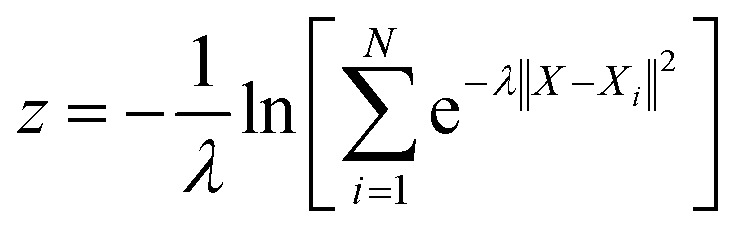
where *N* is the number of reference structures and *X* is the structure described by (C_O_2__–_O_) and (C_O_2__–_H_). The parameter *λ* is selected as 0.25. The Gaussians adopted have an initial height of 0.1 eV and a width of 0.05 and 0.1 for *s* and z collective variables, respectively. The metadynamics are carried out at 350 K, employing a bias factor of 5 and a deposition rate of 125 fs (every 500 steps). For every metadynamics run (except for O_2_ migration as O_2_ is metastable in bulk water), the system is first equilibrated for 0.5 ns.

### 2.6 Training neural network potentials

The NNP ensemble we used for production consists of five neural network potentials with different architectures of the polarizable atom interaction neural network (PaiNN) model.^43^ In this model, all the atoms in a given configuration are treated as nodes in a graph and the information of their connections will be collected and processed by a message function, which will then be passed to an update function for updating node features. After several message passing iterations, the node features will be used as the input of a multilayer perceptron to get its atomic energy or other scalar properties. By summing up the atomic energies of a given structure, we can get its potential energy and forces by calculating the negative derivatives of energy to atomic coordinates. The model can automatically learn the relationship between chemical properties and the positions of atoms by optimizing several hundreds of thousands of model parameters in message and update layers. In contrast, only a few hyperparameters need to be selected (the size of node features, the number of message passing layers, loss ratio of energy and forces, the cutoff radius for collecting distance information of atoms, *etc.*), avoiding the need to manually select and test handcrafted features like Behler–Parrinello symmetry functions.^38,39^ Besides, the model uses both scalar and vector node features to realize rotational equivariance of directional information (*e.g.*, forces) in the graph, providing better prediction of forces.

Table S2† reports the architectures of five models constituting our NNP ensemble and their error metrics after training on the same dataset for up to 1 000 000 steps. These models use different node feature sizes and the number of message-passing layers to induce model diversity, while their cutoff radii are all set to 5 Å. Both the model training and subsequent production MD (MetaD) simulations are conducted on an NVIDIA GeForce RTX 3090 GPU with float32 precision. The weight parameters in these models are randomly initialized and then optimized on the same data split using stochastic gradient descent to minimize the mean square error (MSE) loss, which can be expressed as:9
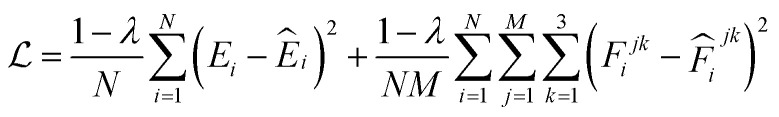
where *N* is the number of configurations, *M* is the number of atoms in a configuration, and *λ* is the force weight that controls the relative importance between energy and force loss. Here the force weight is set to 0.99 as our tests show that using a relatively large force weight can well improve the force prediction while only slightly undermines the precision of energy prediction. Our model parameters are trained by the Adam optimizer^75^ as implemented in PyTorch^76^ with an initial learning rate of 0.0001, the default parameters *β*_1_ = 0.9 and *β*_2_ = 0.999, and a batch size of 16. An exponential decay learning rate scheduler with a coefficient of 0.96 is used to adjust the learning rate for every 100 000 learning steps. The dataset is split into a training set (90%) and a validation set (10%), where the validation set is used for early stopping when the error of forces is small enough. Note that several different error metrics are used to evaluate the performance of the trained model, including mean absolute error (MAE) and root mean squared error (RMSE) for both energy and force predictions. These error metrics can be expressed as follows:10
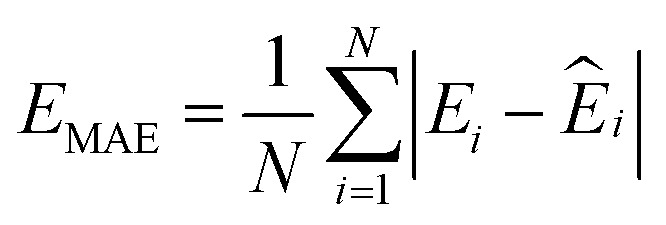
11
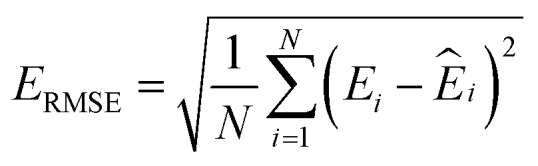
12
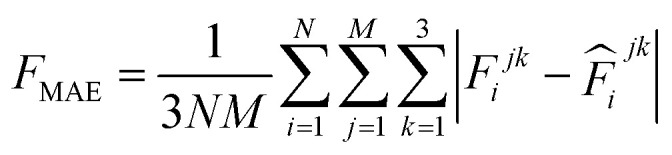
13
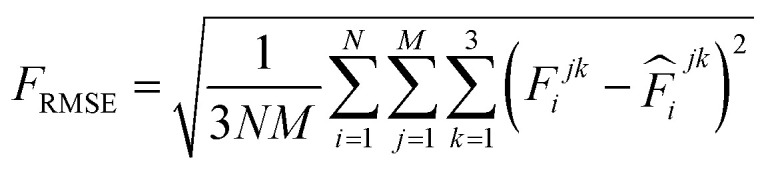


## 3 Results and discussion

### 3.1 Validation of models

**Fig. 2:**
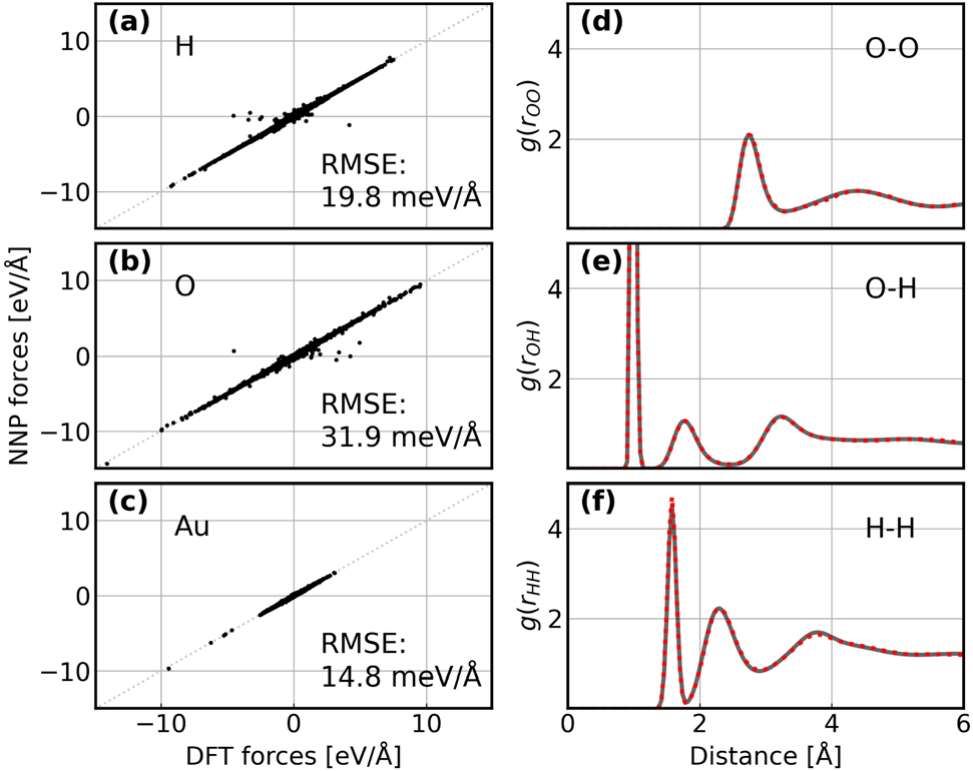
(a–c) Comparison between forces derived from DFT calculations and NNP predicted forces for H, O, and Au. The RMSE of forces for each element are denoted inside. (d–f) Comparison between RDFs obtained from AIMD simulations and NNP MD simulations on the Au(100)–water interface structure. The red points denote RDFs generated by AIMD calculation and the grey solid line denotes RDFs generated by NNP calculation.

Following the active learning framework, we have obtained a final dataset with 18 731 configurations. Fig. S2† shows the learning curves of our NNPs trained on the final dataset, and Table S2† reports the detailed error metrics of best models on the validation set. It is remarkable that our NNPs exhibit exceptional accuracy towards the prediction of energy and forces, where the mean absolute errors (MAEs) of energy range between 0.4 and 0.8 meV per atom, and the MAEs of forces between 12.6 and 16.3 meV Å^−1^. To illustrate the performance of our models on different interface structures, we also report the composition of the final dataset and corresponding error metrics for different structures as shown in Table S3.† The precision of force predictions for each species in our research system is also shown in Fig. 2a, indicating close numerical agreement with DFT results. All these results suggested that the trained NNPs can provide accurate energy and force predictions for different structures across the ORR configurational space in the production MD simulations. Table S4† exhibits the comparison of model performance in terms of energy and force predictions between our model and other studies for complex systems, illustrating that our model outperforms most of these studies, especially force predictions.^43,44,77–80^ The role of accurate force prediction is emphasized in our study since it is critical in MD simulations. The performance of the trained NNP ensemble is further validated in terms of its ability to reproduce the structural properties of AIMD trajectories. Fig. 2b shows the match of the radial distribution functions (RDFs) of all involved species in the case of the Au(100)–water interface (without hydroxyls or oxygen molecules). Apparently, the RDFs generated by NNP MD simulations (solid black line) exhibit excellent agreement with AIMD results (red points), indicating that the NNP ensemble captures the structural arrangement of the gold–water interface well. Apart from validating NNPs with the existing dataset, a more important assessment for the quality of NNPs is their application domain, which can be confirmed by uncertainty measurements. Concretely, the MD runs should be ergodic to ensure the reliability of information derived from them, which indicates that all energetically relevant states must be sampled and within the manifold accessible by NNPs. For all MD simulations in this study, we not only sample the properties of interest along long-time scale MD simulations but also present the uncertainties of all steps by calculating the variance of NNPs. The low force uncertainty of MD simulations for different interface structures verifies the robustness and reliability of the trained NNP ensemble in the given configurational space (the energy and uncertainty profiles in Fig. S3 to S10†). The agreement of the density profiles of water between AIMD and NNP MD with the same box size (3 × 3) is reported in Fig. S11.† It can be observed that the density profile of water in NNP MD simulation is more smooth than that in AIMD, and some disagreements are exhibited in the bulk water area. We ascribe the disagreements and the fluctuation of AIMD density profiles to the inadequate equilibration of AIMD simulations. Moreover, the average energy profiles of Au(100)–water with four *OH that started from different points are well converged as shown in Fig. S12,† indicating that our MD simulations are ergodic and the time scale is long enough.

Except for the validation of model accuracy and reliability, we also evaluated the overall computational efficiency of the proposed scheme in terms of training the initial model, model retraining, CUR matrix decomposition, production MD simulations for 5 ns and DFT labelling as demonstrated in Fig. S13.† For the systems in this study, the required computational time for 1000 AIMD steps is approximately 650 CPU hours, corresponding to 1543.8 hours in total for generating the initial AIMD dataset if using 80 CPU cores for each job. Training on the initial dataset takes about 40 hours, while the cost of retraining the new models can be substantially reduced by loading pretrained model parameters. The MD simulation driven by NNPs accounts for the highest computational cost in an active learning iteration, which takes approximately 7 days to run 5 ns simulations on an NVIDIA RTX3090 GPU. In comparison, AIMD needs more than 7 years to run 5 ns using 80 CPU cores, being about 3–400 times slower than NNP MD. To train the NNPs for a system to run more than 5 ns MD, 5 to 10 iterations are usually needed, which corresponds to 1000–2000 labelled structures as indicated in Table S3.† It is worth noting that the ASE MD engine used in this study is not specialized for GPU computing, resulting in high overheads of data transfer between the GPU and CPU. It can be expected that the computational efficiency of NNP MD can be further improved in the future by using a GPU-specialized MD code.

**Fig. 3:**
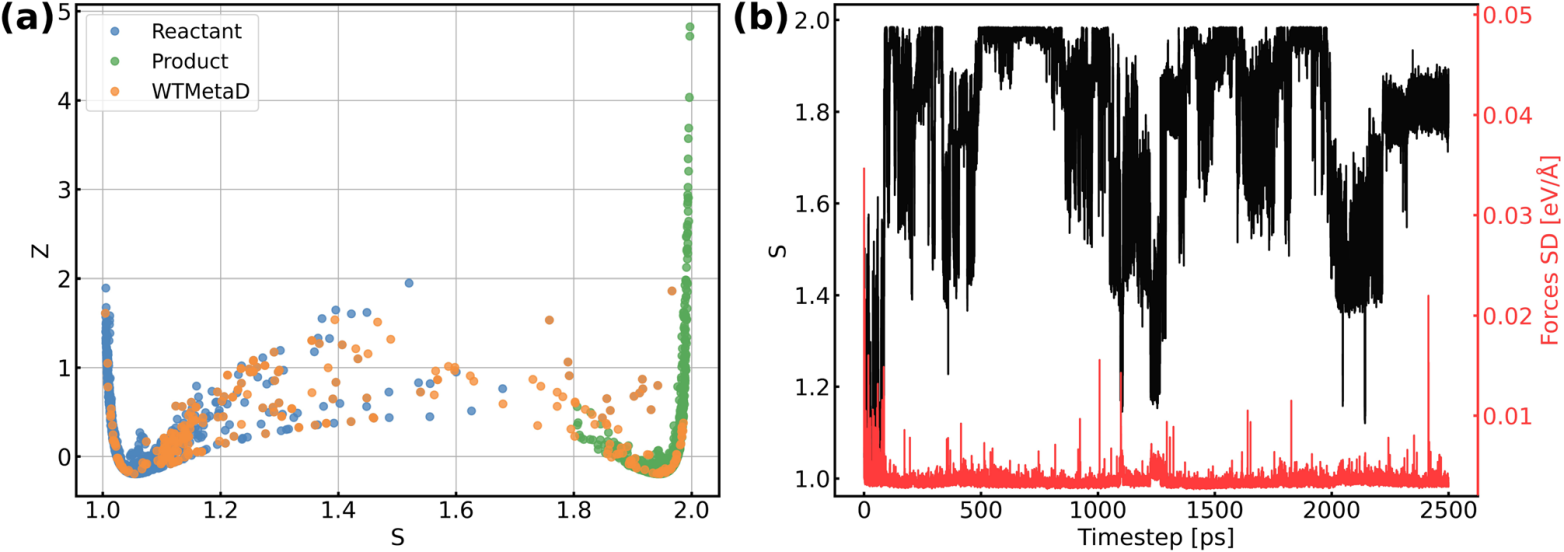
(a) Distribution of reference structures in the configurational space described by path collective variables *s* and *z*, where *s* represents the progress along the path between reactants and products and *z* represents the distance from the path. (b) Evolution of *s* and force standard deviation (SD) along 2.5 ns MetaD simulation.

The validation of NNPs *via* application in MetaD simulations is crucial as the configurational space of the full reactive process can be huge while the transitional states are rarely visited. As shown in Fig. 3, our training data points are evenly distributed in the configurational space described by path collective variables,^81^ and the force uncertainties along 2.5 ns MetaD simulations are all considerably small (all smaller than 0.05 eV Å^−1^). Both metrics build confidence that the trained NNPs are reliable to capture the characteristics of all energetically relevant states, especially transitional states, of the ORR. Furthermore, the trained models have shown excellent transferability when using them for the inference of Au(110)–water and Au(111)–water interfacial systems as demonstrated in Fig. S1a.† Despite missing structural information for the two similar systems, the trained models still well predicts the energy and forces with both low errors and uncertainties for all Au(110)–water and Au(111)–water interfacial structures, which indicates that the proposed scheme and trained models can easily generalize to systems across a wide range of metals and their different facets.

### 3.2 Full metadynamics simulation of the ORR

After systematic validations, the trained NNPs are used to study both adsorption energetics and kinetics of the ORR at the gold–water interface. It is well known that the ORR on Au(100) in alkaline electrolytes proceeds *via* the complete four-electron transfer mechanism, while the partial two-electron transfer mechanism dominates on other Au facets, such as Au(111) and Au(110).^17,55,56^ Despite the use of new techniques and persistent efforts devoted by researchers, the reason why ORR activity is exceptional and facet-dependent on gold remains elusive. There are several assumptions that may provide a clear answer to this question, including the outer-sphere mechanism of the ORR,^17,82,83^ and the role of preadsorbed species and solvents.^84,85^ All these assumptions call for a full atomic simulation that elaborately considers the ambient conditions of Au(100) and models the reaction without any simplification.

**Fig. 4:**
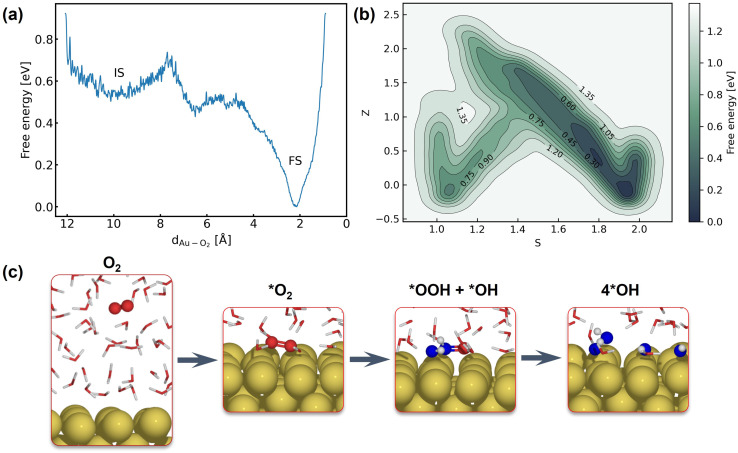
(a) Free energy landscape of O_2_ migration from bulk water to the Au(100) surface. (b) Free energy landscape of *O_2_ reduction to *OH described by path collective variables. (c) Snapshots for O_2_ in bulk water, the initial state, the transitional state, and the final state.

The first step of the ORR on Au(100) is O_2_ activation, which is also considered a key step that determines the activity of catalysts that weakly interact with adsorbates. According to whether O_2_ closely adsorbs on Au(100), the reaction can be initiated *via* the inner-sphere mechanism in which the slab directly transfers electrons to closely adsorbed O_2_, or the outer-sphere mechanism in which the ORR occurs away from the slab by several solvent layers. The adsorption energy of the *O_2_ molecule and *OH with different coverage is summarized in Table S5,† suggesting weak interaction between these species and the Au(100) slab. As demonstrated in Fig. S9,† our 5 ns MD simulations at the Au(100)–water interface with one O_2_ molecule have shown that the O_2_ molecule will be in close contact with the Au(100) surface, yielding a density peak at 2.1 Å. We further carried out a MetaD simulation that models the migration of O_2_ from bulk water to the Au(100) surface as shown in Fig. 4a. It is found that there are no stable local minima for O_2_ saturating in bulk water, and the migration barrier can be easily overcome by the thermal fluctuation of the model system. As shown in Fig. S14,† a simple MD simulation modeling the movements of O_2_ in the bulk water part of the interface also proves this conclusion. After 350 ps simulations, the O_2_ molecule finally moved from bulk water to the Au(100) surface. Based on these results, we model the reaction process with O_2_ directly adsorbed on Au(100) and believed that the bond breaking of the O_2_ molecule could be the rate-determining step of the ORR on Au(100).

The full atomic simulation of the ORR is then conducted to investigate the bond-breaking process in the O_2_ molecule and the formation of hydroxyls by using metadynamics simulations. The reaction coordinates of the ORR are described by path collective variables (CVs)^81^ with the initial state (Au(100)–1O_2_/57H_2_O) and final state (Au(100)–4OH/55H_2_O) selected as two reference structures. The distance to reference structures is quantified by the number of oxygen atoms (C_O_2__–_O_) and hydrogen atoms (C_O_2__–_H_) around the O_2_ molecule. As summarized in Table S6,† these two descriptors can well capture and differentiate the structural characteristics of different possible intermediate states of the ORR, including *O_2_, *OOH, *H_2_O_2_, *O, and *OH. The well-designed CVs enable us to automatically search the reaction path without using any prior knowledge about the reaction mechanism. In this approach, instead of modeling multiple possible reaction pathways and verifying which one is energetically most favorable, we only need to incrementally extend the explored PES (with our NNPs) from equilibrium states to non-equilibrium transitional states by using active learning. Furthermore, this strategy can be easily generalized to simulate more complex model systems and chemical reactions.

Fig. 4b shows the obtained free energy landscape of the ORR as a function of path CVs, where *s* is the progress along the reference path, and *z* is the distance to the reference path. The landscape is composed of two basins which correspond to the initial state and final state of the ORR. Fig. 3b also shows the time evolution of the *s* collective variable. It can be seen that the first basin in the landscape has been completely filled after approximately 100 ps, which corresponds to the transition from O_2_ to hydroxyls. Filling the second basin, which can be regarded as the transition from hydroxyls to O_2_, becomes much more difficult than the first one with the employed CVs in this study. However, it should be pointed out that the depth of the first basin is enough to evaluate the activation energy of bond breaking in O_2_. The energy barrier of the transition from O_2_ to hydroxyls is estimated to be 0.3 eV, which is in good agreement with experimental findings that Au(100) displays high ORR activity. It is noteworthy that the simulation box in this study is small in comparison with the realistic interface structure. The limited cell size can result in slightly higher formation energies of hydroxyl as demonstrated in Table S5,† which can be ascribed to the stronger repulsion between hydroxyl in smaller boxes and the possible lateral correlation of solvation shells. Besides, we also expect that the bond breaking of the O_2_ molecule can be more difficult because of the easier recombination of individual oxygen atoms. Both effects can make the ORR in a small cell less facile, while further supporting our conclusion that the ORR is facile on Au(100) even when modeled with a limited number of water molecules. The snapshots for O_2_ in bulk water, the initial state, the transition state, and the final state are displayed in Fig. 4c. At first, the O_2_ molecule is partially protonated by neighboring water molecules to *OOH, suggesting the associative reaction pathway proposed by Nørskov *et al.*^1^ However, the subsequent formation of *O is not observed in the overall reaction as the remaining oxygen atom is immediately protonated by reacting with water. Therefore, the reaction pathway observed from our simulations can be summarized as follows:*O_2_ + H_2_O → *OOH + *OH*OOH + H_2_O → 3*OHThe MetaD simulation highlights the role of water molecules as a reactant of the ORR, suggesting that the explicit modeling of solvents is indispensable in theoretical electrocatalysis.

## 4 Conclusions

In summary, the reactive process of the ORR is investigated by MetaD simulations that are significantly accelerated by high fidelity NNPs in this study. By using an active learning strategy underpinned by CUR matrix decomposition, we obtained an NNP ensemble that exhibits exceptional performance and reliability for the prediction of structural properties and forces in the configurational space of an Au(100)–water interface. By leveraging well-designed path collective variables, the ORR can be fully and automatically simulated without the need to elaborately consider multiple reaction pathways. Our MetaD simulations suggest that the ORR proceeds in the associative reaction pathway, while the *OOH reaction intermediate is directly reduced to two *OH with the participation of neighboring water molecules rather than dissociating into *OH and *O. The low energy barrier of the ORR predicted in this study well explains the outstanding experimental ORR activity. The longer time-scale simulations enabled by NNPs can give us deeper insight into the nature of chemical reactions, such as the facet-dependent ORR on different Au facets which will be pursued in our future work. Besides, the effect of cations on the ORR activity of gold is also a meaningful extension of this work. The full atomic simulation conducted here can be conveniently extended to other model systems and become a valuable tool for investigating complex chemical reactions in a straightforward manner.

## Data availability

The code for training the PaiNN model, performing MD and MetaD simulations, and CUR matrix decomposition is available in the following GitHub repository: https://github.com/Yangxinsix/painn-sli. The dataset in this study is openly available in the DTU data repository.^86^

## Author contributions

X. Y. wrote the code, ran the calculations, analyzed the results, and wrote the original draft. X. Y., A. B., and H. A. H. conceived the research. A. B. and T. V. and H. A. H. supervised the research and helped revise the manuscript. All authors discussed and commented on the manuscript.

## Conflicts of interest

There are no conflicts to declare.

## Supplementary Material

SC-014-D2SC06696C-s001

SC-014-D2SC06696C-s002
