# Supplementary material for: Neural network potentials for accelerated metadynamics of oxygen reduction kinetics at Au–water interfaces
Source: Chem Sci. 2023 Mar 13;14(14):3913–22. doi: 10.1039/d2sc06696c (PMC10074416; doi:10.1039/d2sc06696c)
Supplement: SC-014-D2SC06696C-s002 [file SC-014-D2SC06696C-s002.pdf]

Electronic Supplementary Material (ESI) for Chemical Science.
This journal is © The Royal Society of Chemistry 2023

Neural Network Potentials for Accelerated Metadynamics of Oxygen Reduction Kinetics at Au-Water Interfaces

Xin Yang, Arghya Bhowmik, Tejs Vegge, and Heine Anton Hansen*

Department of Energy Conversion and Storage, Technical University of Denmark, Anker Engelunds Vej, 2800Kgs. Lyngby, Denmark

February 22, 2023

1 Supplementary Tables

Table S1: Parameters for calculating coordination numbers of O_2

CV	Definition	Parameters
C_{O_2-O}	$C_{O_2-O} = \sum_{i \in O_2} \frac{1-\left(\frac{r_{i,O}-d_0}{r_0}\right)^n}{1-\left(\frac{r_{i,O}-d_0}{r_0}\right)^m}$	$r_{i,O}$: distance between O_i and O<br>$r_0 = 1.8, d_0 = 0, n = 6, m = 12$
C_{O_2-H}	$C_{O_2-H} = \sum_{i \in O_2} \frac{1-\left(\frac{r_{i,H}-d_0}{r_0}\right)^n}{1-\left(\frac{r_{i,H}-d_0}{r_0}\right)^m}$	$r_{i,H}$: distance between O_i and H<br>$r_0 = 1.5, d_0 = 0, n = 8, m = 16$

Table S2: A summary of test error metrics of neural network potentials

Model	Node size	Layers	Energy error (meV/atom)		Forces error (meV/Å)			
			MAE	RMSE	l_2 MAE	l_2 RMSE	MAE	RMSE
NNP1	96	3	0.6	1.3	32.8	46.4	16.3	26.8
NNP2	112	3	0.5	1.3	31.3	45.0	15.5	26.0
NNP3	128	3	0.8	1.4	28.9	43.7	14.3	25.2
NNP4	128	4	0.4	1.2	25.4	39.3	12.6	22.7
NNP5	144	3	0.5	1.2	27.0	40.8	13.4	23.5
Ensemble	-	-	0.7	1.4	25.3	38.8	12.6	22.4

Table S3: A summary of interface structures presented in the final dataset after CUR selection

Interface structure	Number of atoms			Number of configurations			E_{MAE} (meV/atom)	F_{MAE} (meV/Å)
	H	O	Total	Training	Validation	Total		
Au(100)-30H_2O	60	30	126	616	70	686	2.3	14.2
Au(100)-1OH/29H_2O	59	30	125	1535	172	1707	1.4	12.8
Au(100)-2OH/28H_2O	58	30	124	646	74	1694	1.1	14.0
Au(100)-1O_2/30H_2O	60	32	126	1192	136	1328	1.6	13.8
Au(100)-1OH/58H_2O	117	59	240	1458	155	1613	0.7	11.6
Au(100)-2OH/57H_2O	116	59	239	1541	153	1694	0.6	11.6
Au(100)-3OH/56H_2O	115	59	238	1667	209	1876	0.5	12.1
Au(100)-4OH/55H_2O	114	59	237	2188	245	2433	0.3	12.4
Au(100)-5OH/54H_2O	113	59	236	2110	232	2342	0.3	12.9
Au(100)-6OH/53H_2O	112	59	235	1986	229	2215	0.3	13.8
Au(100)-1O_2/57H_2O	114	59	237	1826	188	2014	0.4	12.0

Table S4: Comparison of model performance between previous studies and this work

Ref.	System	Method	Max. N_{atom}	Training set	Errors
Natarajan et al.[1]	Cu-H_2O	BPNNP	463	10293 structures with bulk water/ice, bulk copper/cuprous oxide and water–copper interface geometries. 10% are used for validation.	E_{RMSE}: 0.9 meV/atom<br>F_{RMSE}: 125.3 meV/Å
Quaranta et al.[2]	ZnO-H_2O	BPNNP	334	15319 structures with bulk water, bulk ZnO, and ZnO-water interface geometries. 1712 configurations are used for validation.	E_{RMSE}: 1.2 meV/atom<br>F_{RMSE}: 143.4 meV/Å
Yang et al.[3]	Urea-water	DeepMD	110	14536 structures with 5739 reactant structures, 5217 product structures, and 3580 transition state structures.	E_{MAE}: 0.7 meV/atom<br>F_{MAE}: 38 meV/Å
He et al.[4]	$SrTiO_3$	DeepMD	40	2600 structures with $2 \times 2 \times 2$ and $1 \times 1 \times 1$ supercells. A test set with 1500 structures of 80 atoms are used for validation.	E_{MAE}: 0.3 meV/atom<br>F_{MAE}: 19 meV/Å
Liu et al.[5]	βGa_2O_3	GAP	160	801 training structures obtained from MD simulations at temperatures between 100 K and 1000 K. 90 structures are used for validation.	E_{RMSE}: 0.5 meV/atom<br>F_{RMSE}: 50 meV/Å for Ga<br>F_{RMSE}: 38 meV/Å for O
Davidson et al.[6]	$\alpha Fe - H$	GAP	128	The training data for the H-Fe interaction potential comprises snapshots from molecular dynamics trajectories of 54 and 128 Fe atoms with either 0, 1, or 2 Fe atoms removed, and a single H atom added. Altogether, about 400 configurations were used in the fit, comprising about 28k atoms.	E_{MAE}: 20 meV<br>F_{MAE}: 10 meV/Å
Hu et al.[7]	OC20 dataset[8]	ForceNet	225	OC20 dataset[8] contains 200M+ nonequilibrium 3D atomic structures with average atom number of 73.3 from 1M+ atomic relaxation trajectories. The model is trained on 134M structures from S2F task. Four validation datasets are used to test the model performance: In Domain (ID),Out of Domain Adsorbate (OOD Adsorbate), OOD Catalyst, and OOD Both (both the adsorbate and catalyst's material are not seen in training). Each split contains 1M examples.	F_{MAEs}<br>ID: 28.1 meV/Å<br>OOD Ads.: 32.0 meV/Å<br>OOD Cat.: 32.7 meV/Å<br>OOD Both: 41.2 meV/Å<br>Average: 33.5 meV/Å
Gasteier et al.[9]	OC20 dataset	GemNet-OC	225	The model is trained on $\tilde{1}$34M structures from S2EF task in OC20 dataset. The same test set splits are used as above.	Average:<br>E_{MAE}: 233 meV<br>F_{MAE}: 20.7 meV/Å
Li et al.[10]	Water	GAMD	384	7000 periodic configurations of liquid water. The number of water molecules in the unit cell ranges from 16 to 128. 723 snapshots are used for validation.	F_{MAE}: 24.28 ± 16.80 meV/Å<br>F_{RMSE}: 35.39 ± 23.09 meV/Å
Batzner et al.[11]	$Li_4P_2O_7$	Nequip	208	The crystal structure was melted at 3000 K for 50 ps, and quenched at 600 K for another 50 ps, resulting a dataset of 25,000 AIMD frames. 1000 structures from melting phase are used for training, 100 structures for validation, and all remaining structures for independent test.	Melt(quench):<br>E_{MAE}: 0.4(0.5) meV/atom<br>F_{MAE}: 34.0(21.3) meV/Å<br>E_{RMSE}: 0.8(0.5) meV/atom<br>F_{RMSE}: 59.5(34.9) meV/Å
Ours	Au-water	PaiNN	240	18371 Au(100)-water interface structures with different number of *OH and O_2 presented in the liquid. The rare event structures in metadynamics are also included.	E_{MAE}: 0.7 meV/atom<br>F_{MAE}: 12.6 meV/Å<br>E_{RMSE}: 1.4 meV/atom<br>F_{RMSE}: 22.4 meV/Å

Table S5: Adsorption energies of different species on Au(100) surface

Species	Cell size	θ (Coverage)	$\Delta E/n_{OH}$ (eV)
1OH	(4×4)	0.063	0.717
2OH	(4×4)	0.125	0.795
3OH	(4×4)	0.188	0.862
4OH	(4×4)	0.250	0.925
5OH	(4×4)	0.313	0.958
6OH	(4×4)	0.375	0.987
1O2	(4×4)	0.063	-1.008
1OH	(3×3)	0.111	0.723
2OH	(3×3)	0.222	0.858

Table S6: Coordination numbers of different possible intermediate structures in ORR

CV	$*O_2$	*OOH	*O + *OH	$*O_2H_2$	*OH
C_{O_2-O}	1	1	0	1	0
C_{O_2-H}	0	0.5	0.5	1	1

2 Supplementary Figures

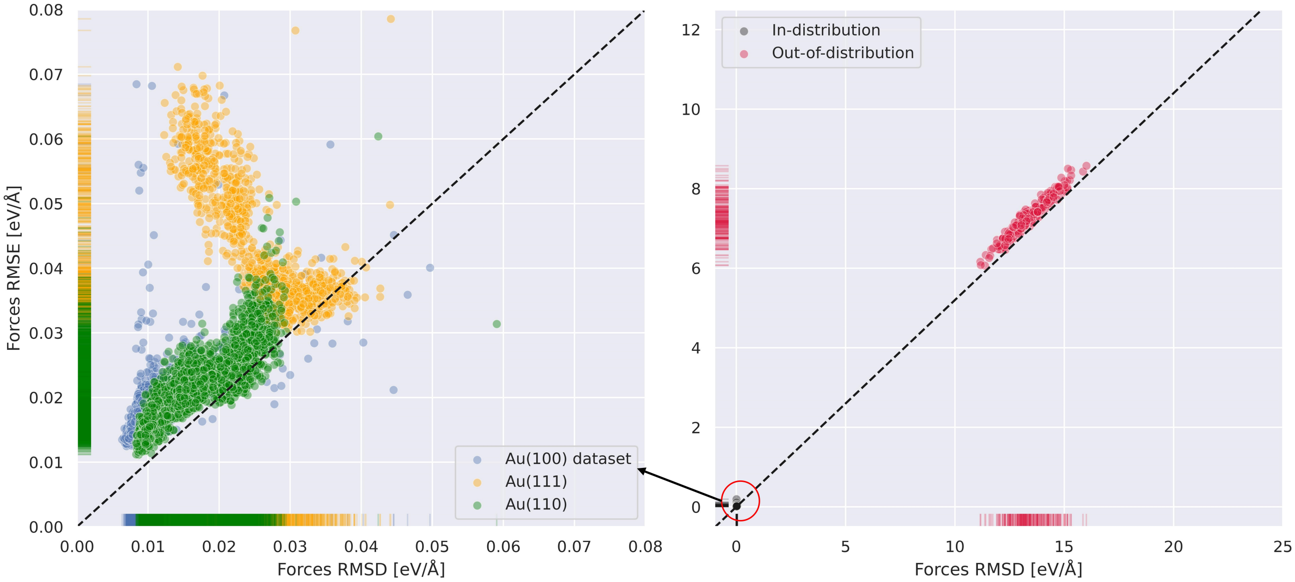

Figure S1: Comparison between ensemble uncertainties calculated by forces root mean square deviation (RMSD) and true prediction error calculated by root mean square error (RMSE) for (a) in-distribution data, and (b) out-of-distribution data.

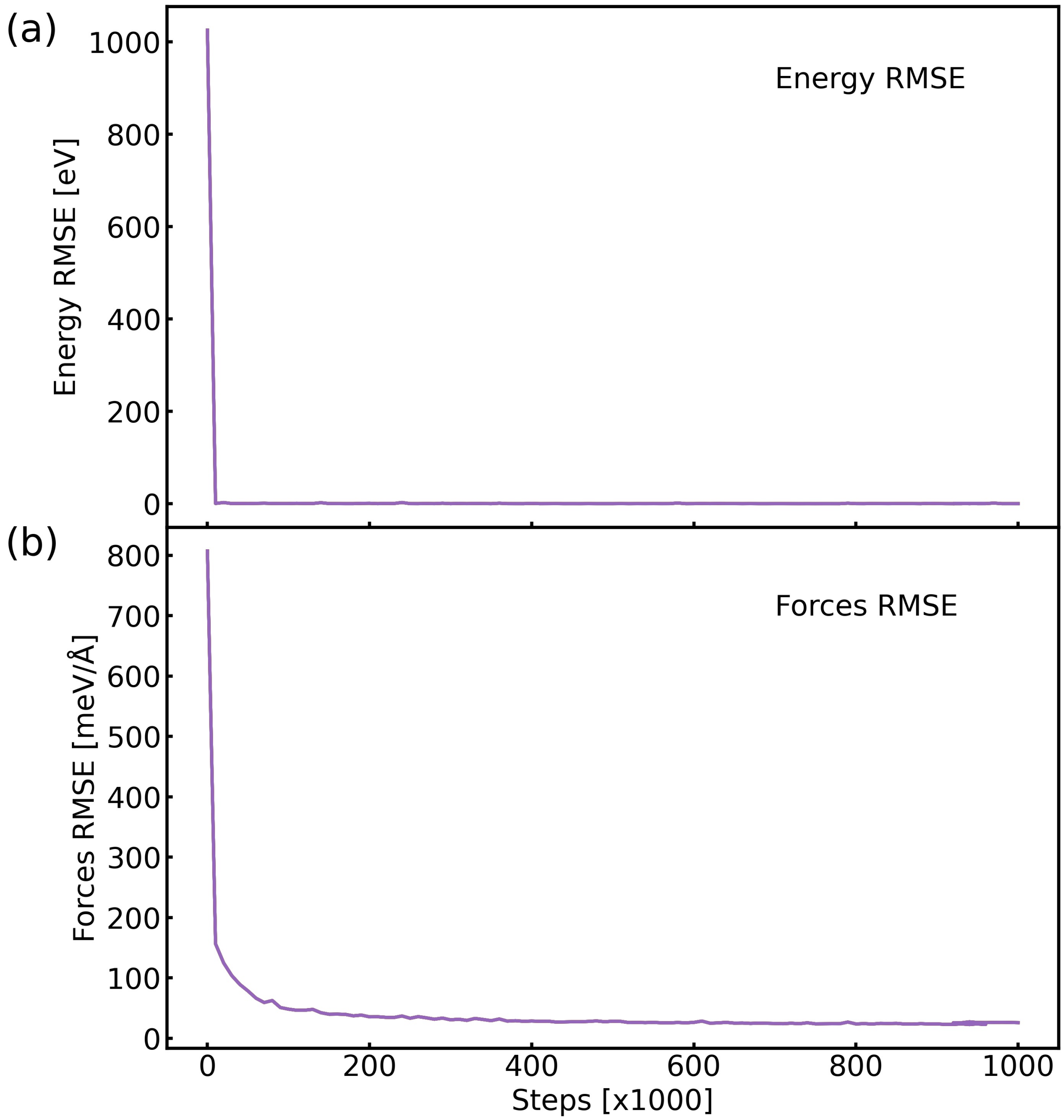

Figure S2: Training curves of the final dataset for (a) energy root mean squared error (RMSE) and (b) forces RMSE

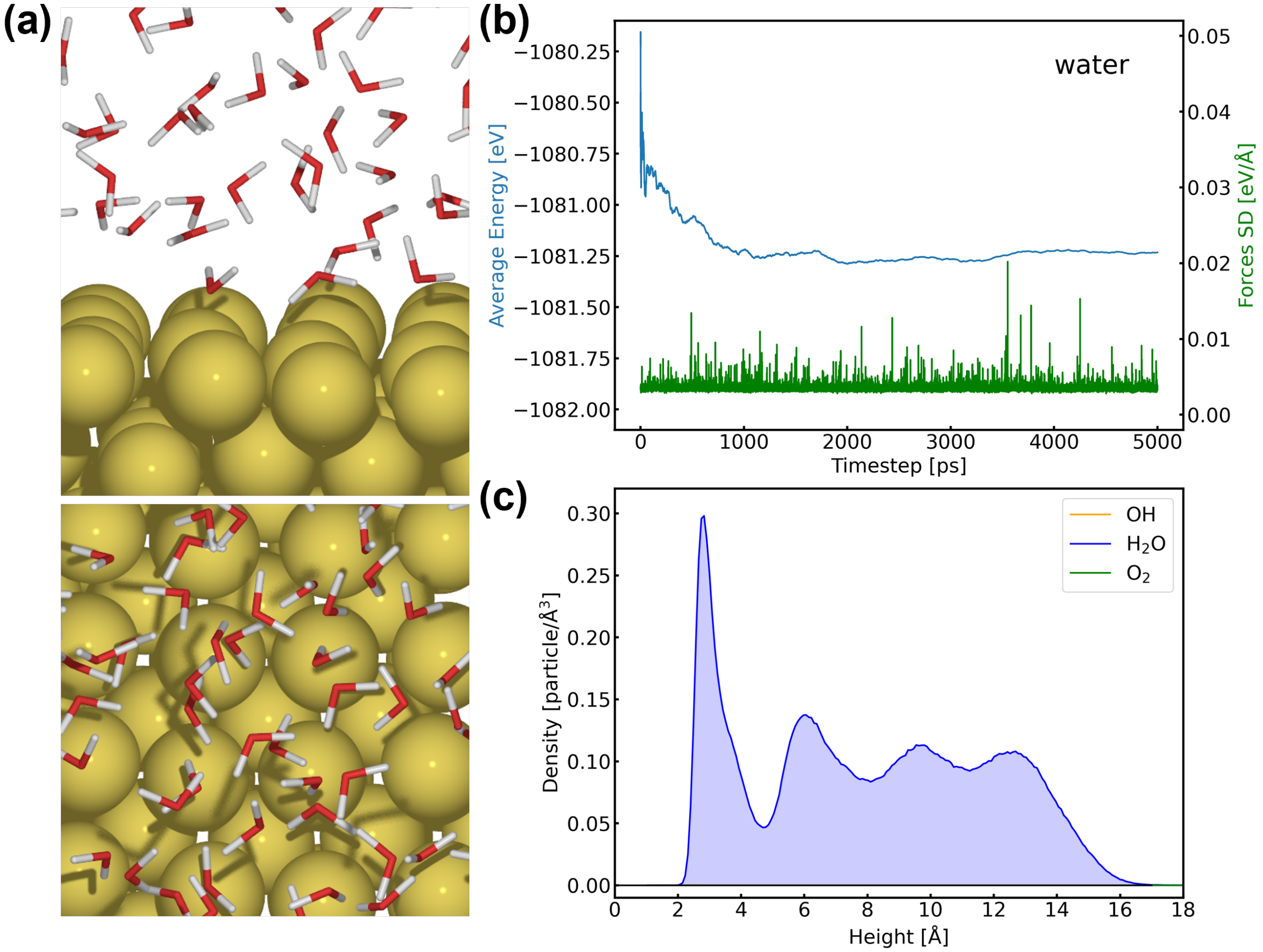

Figure S3: (a) Side view and top view of Au(100)-59H_2O interface structure. (b) Evolution of average energy and force standard deviations (SD) of Au(100)-59H_2O along 5 ns MD simulations. (c) Density profiles of different species as a function of the distance from Au(100) surface.

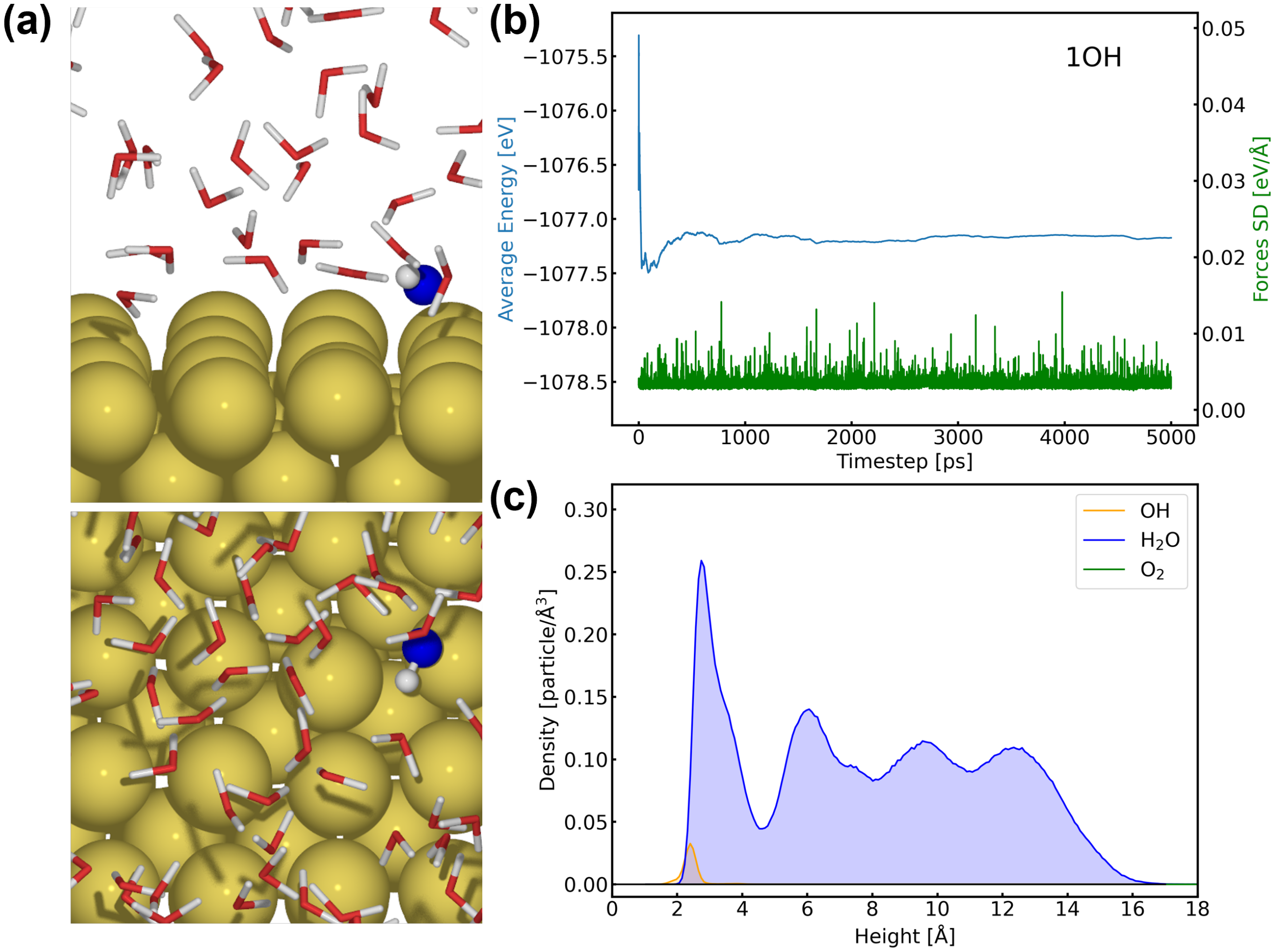

Figure S4: (a) Side view and top view of Au(100)-1OH/58H_2O interface structure. (b) Evolution of average energy and force standard deviations (SD) of Au(100)-1OH/58H_2O along 5 ns MD simulations. (c) Density profiles of different species as a function of the distance from Au(100) surface.

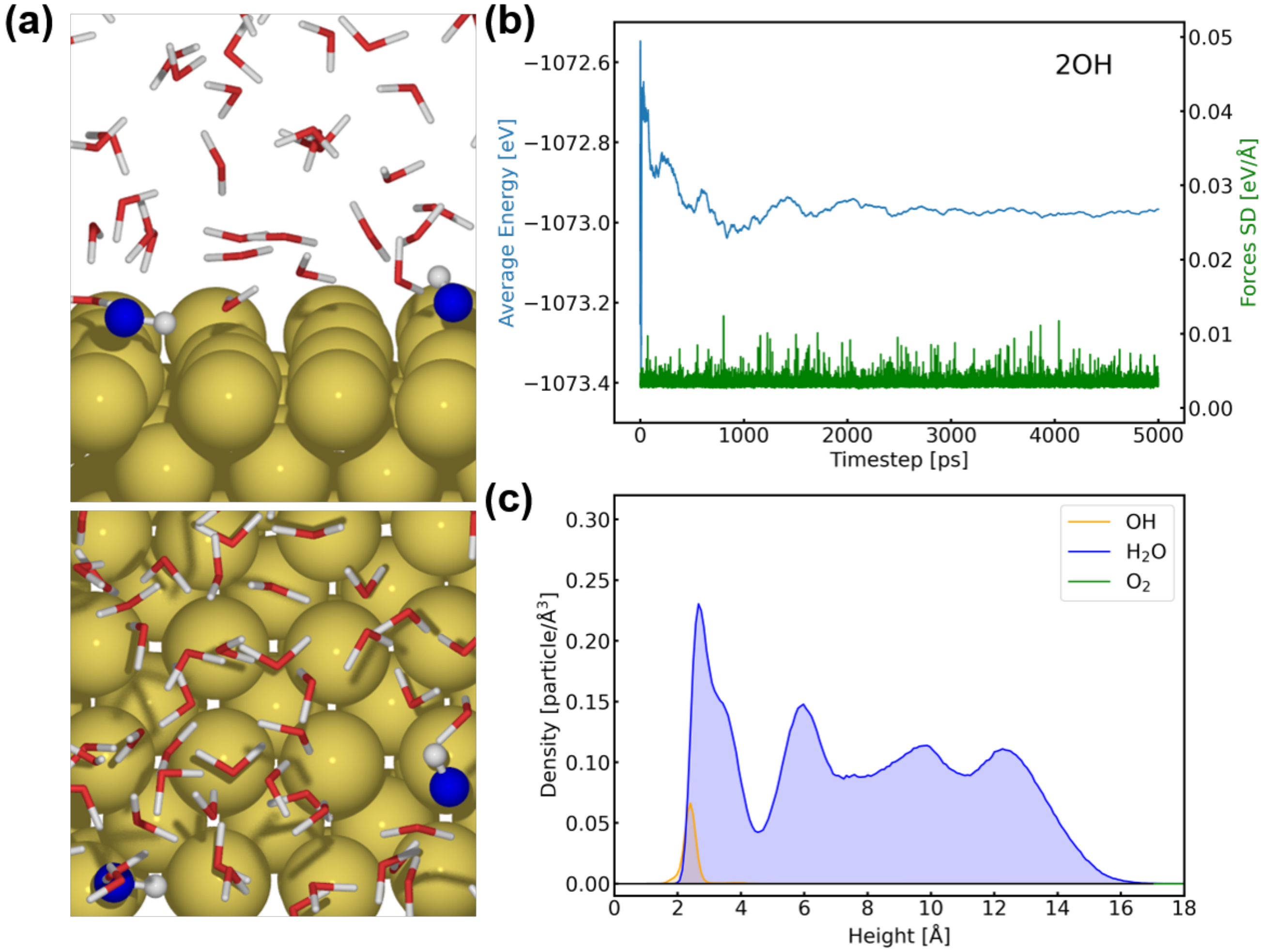

Figure S5: (a) Side view and top view of Au(100)-2OH/57H_2O interface structure. (b) Evolution of average energy and force standard deviations (SD) of Au(100)-2OH/57H_2O along 5 ns MD simulations. (c) Density profiles of different species as a function of the distance from Au(100) surface.

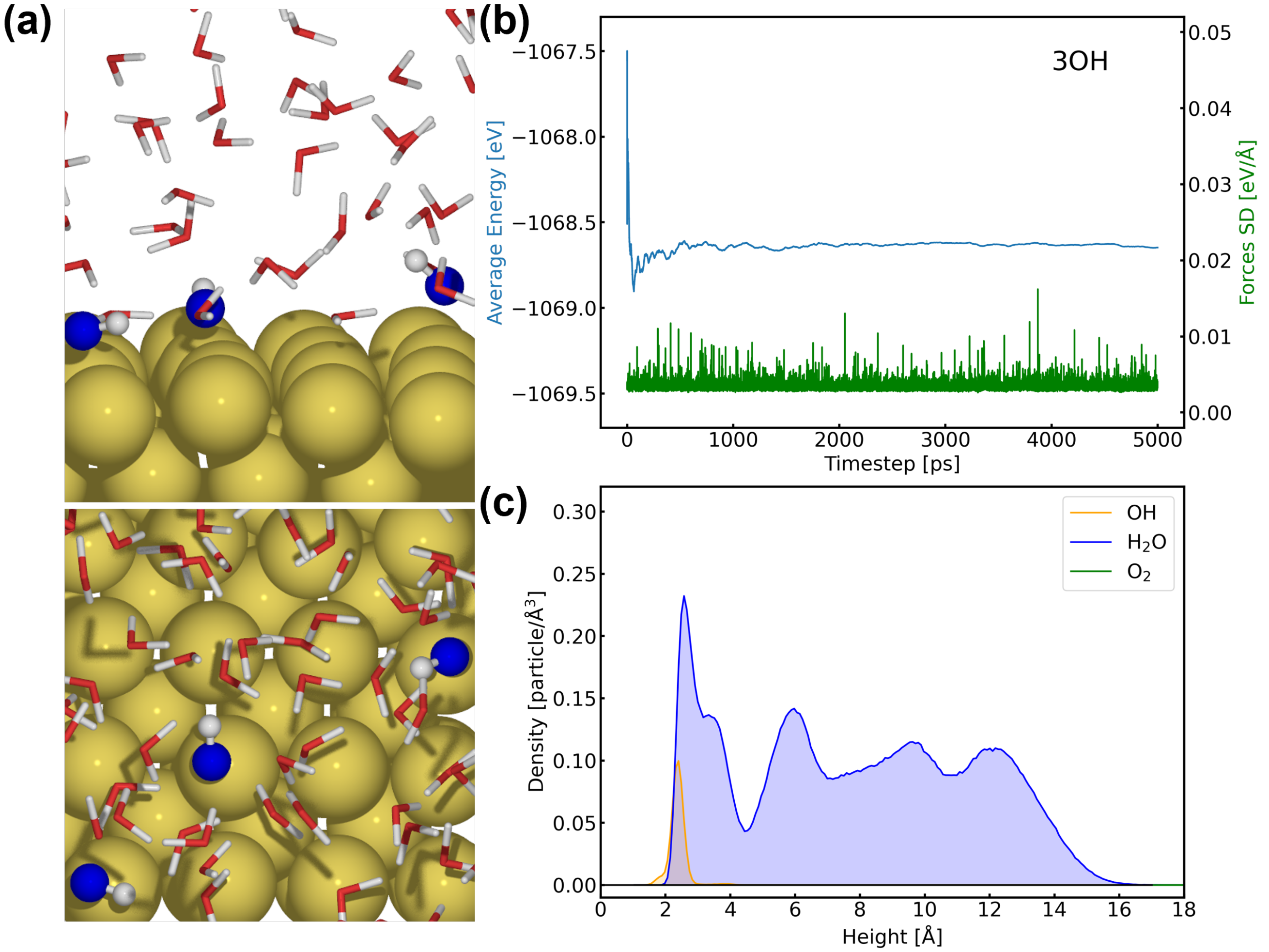

Figure S6: (a) Side view and top view of Au(100)-3OH/56H_2O interface structure. (b) Evolution of average energy and force standard deviations (SD) of Au(100)-3OH/56H_2O along 5 ns MD simulations. (c) Density profiles of different species as a function of the distance from Au(100) surface.

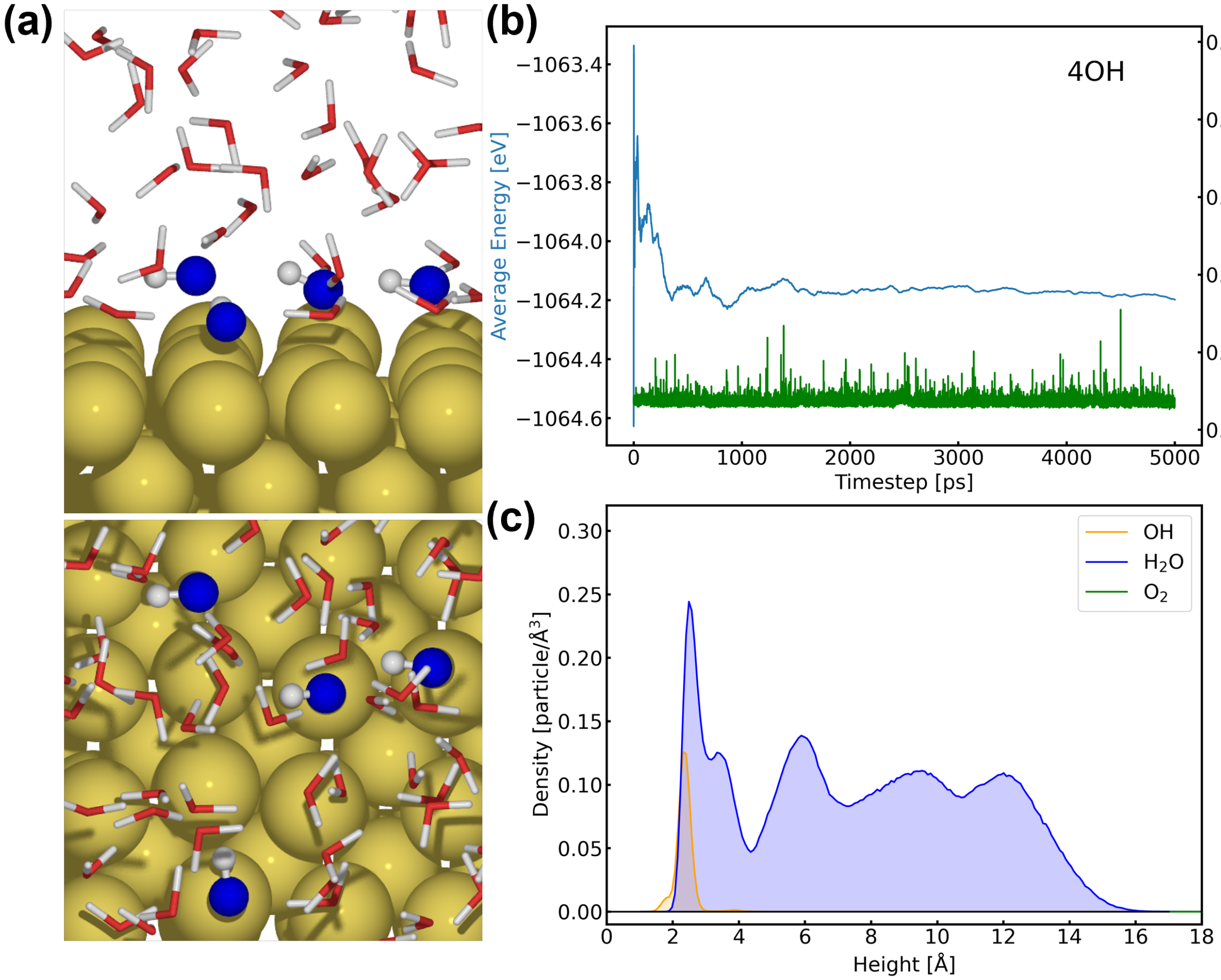

Figure S7: (a) Side view and top view of Au(100)-4OH/55H_2O interface structure. (b) Evolution of average energy and force standard deviations (SD) of Au(100)-4OH/55H_2O along 5 ns MD simulations. (c) Density profiles of different species as a function of the distance from Au(100) surface.

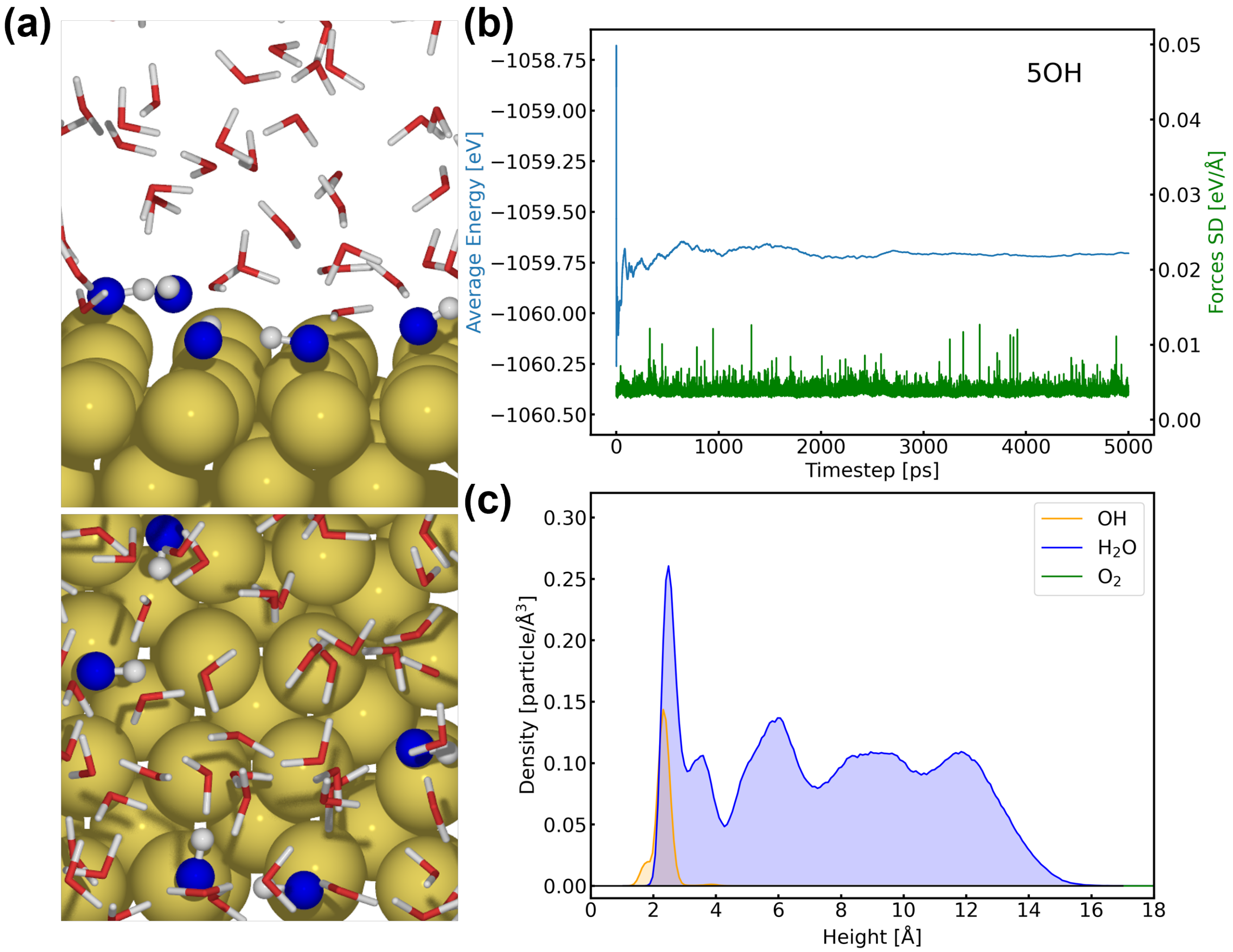

Figure S8: (a) Side view and top view of Au(100)-5OH/54H_2O interface structure. (b) Evolution of average energy and force standard deviations (SD) of Au(100)-5OH/54H_2O along 5 ns MD simulations. (c) Density profiles of different species as a function of the distance from Au(100) surface.

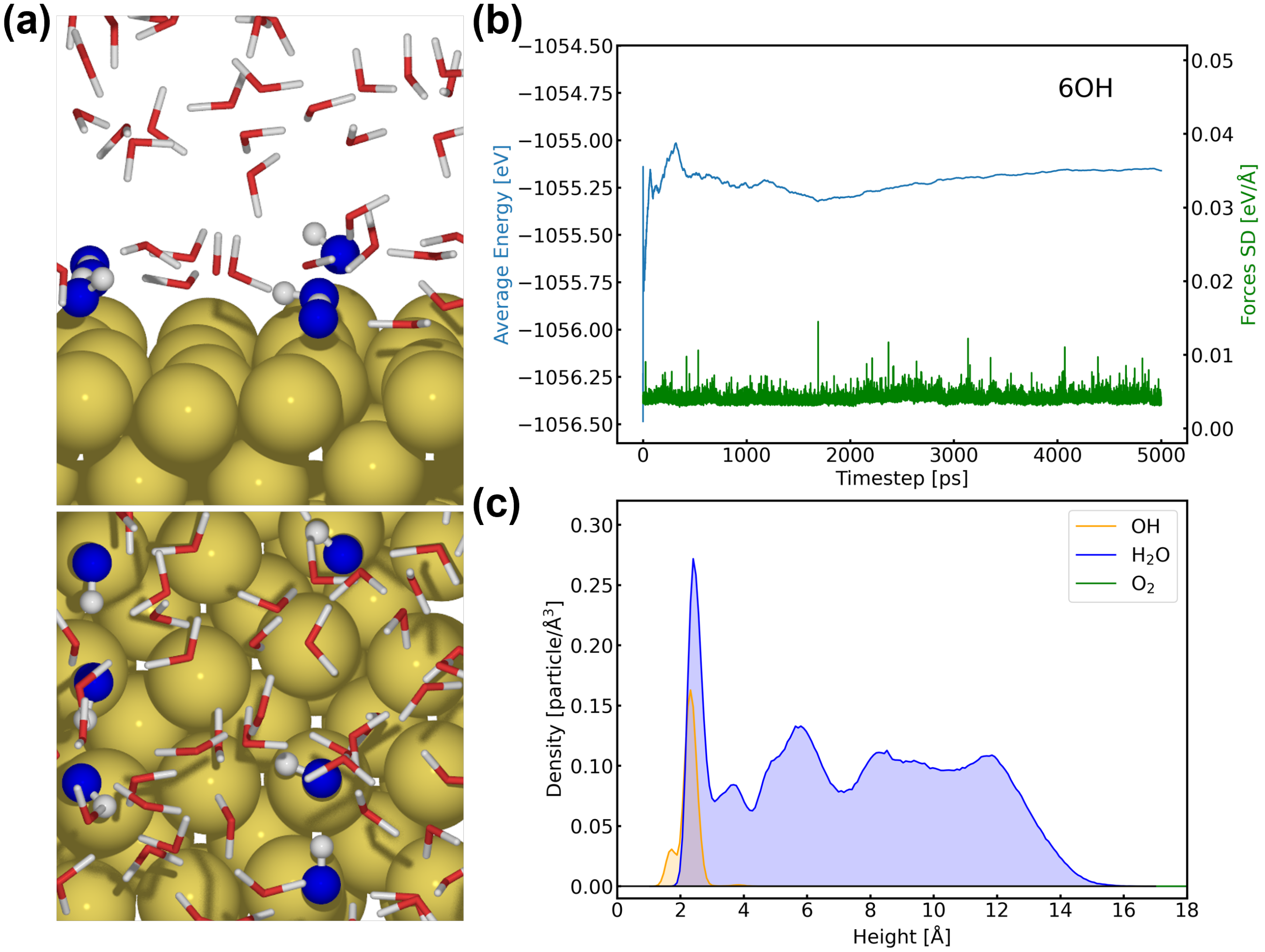

Figure S9: (a) Side view and top view of Au(100)-6OH/53H_2O interface structure. (b) Evolution of average energy and force standard deviations (SD) of Au(100)-6OH/53H_2O along 5 ns MD simulations. (c) Density profiles of different species as a function of the distance from Au(100) surface.

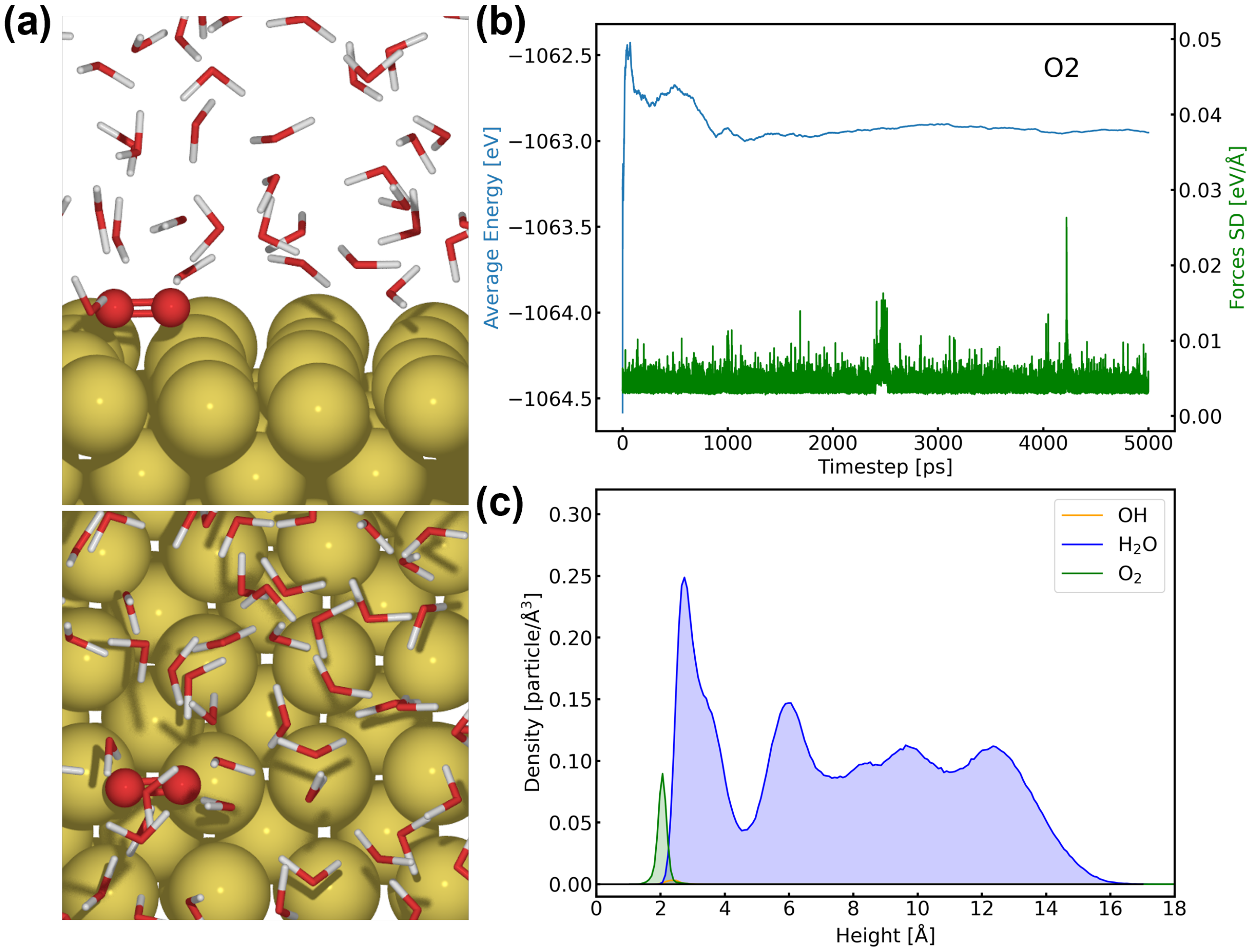

Figure S10: (a) Side view and top view of Au(100)-1O_2/30H_2O interface structure. (b) Evolution of average energy and force standard deviations (SD) of Au(100)-1O_2/30H_2O along 5 ns MD simulations. (c) Density profiles of different species as a function of the distance from Au(100) surface.

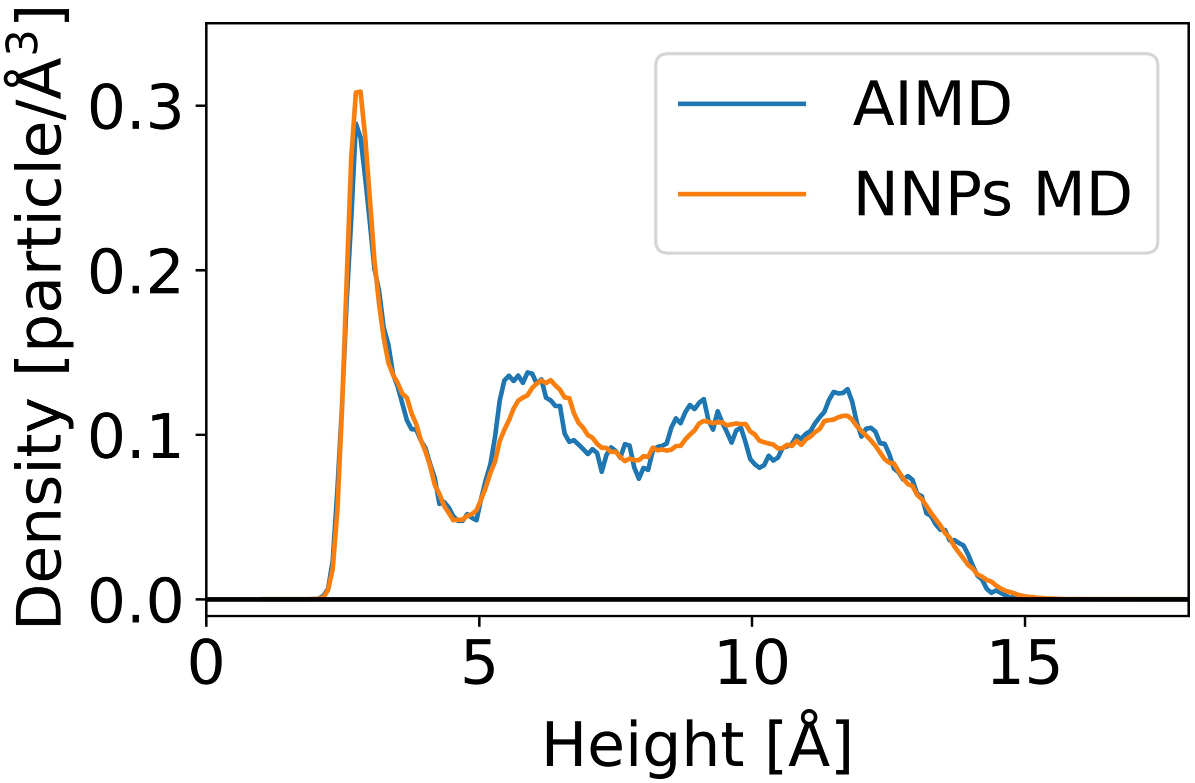

Figure S11: Density profiles of water as a function of the distance from Au(100) surface obtained from 50 ps AIMD (blue) and 5 ns NNPs MD (orange).

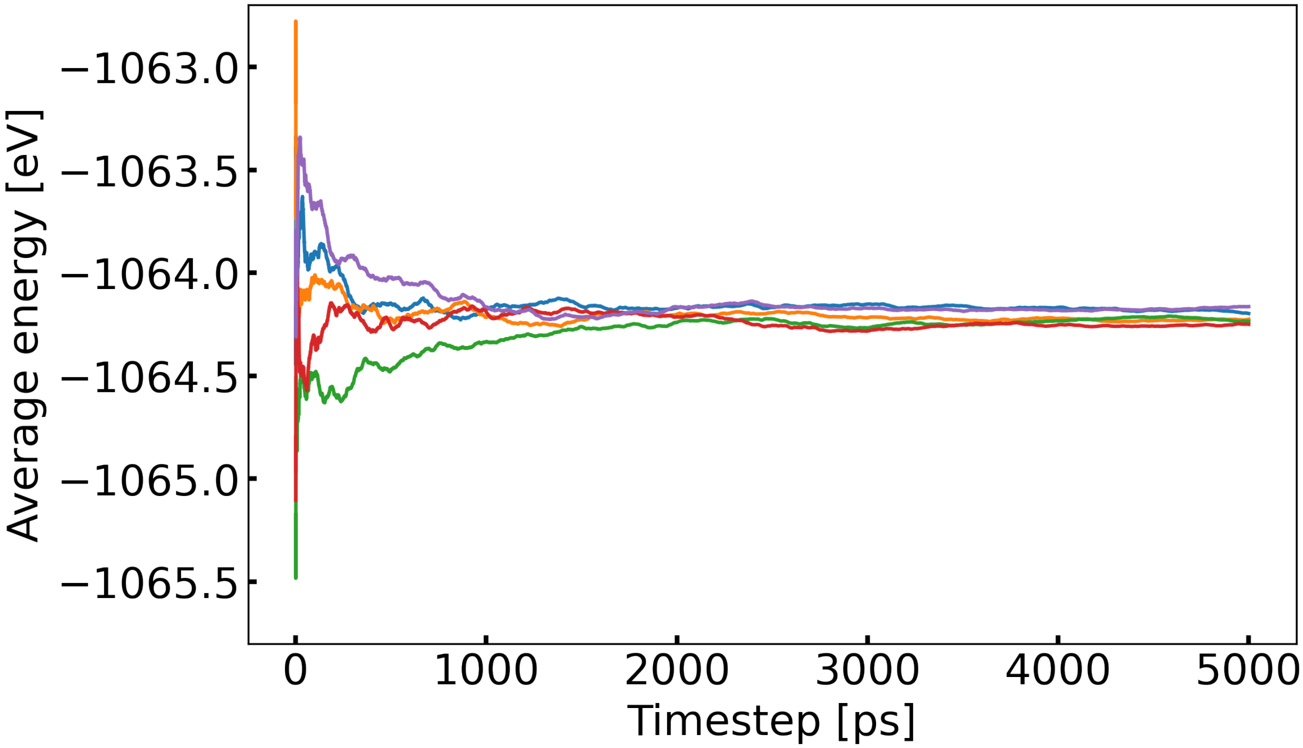

Figure S12: Average energy profiles of Au(100)-4OH/55H_2O from five different starting configurations.

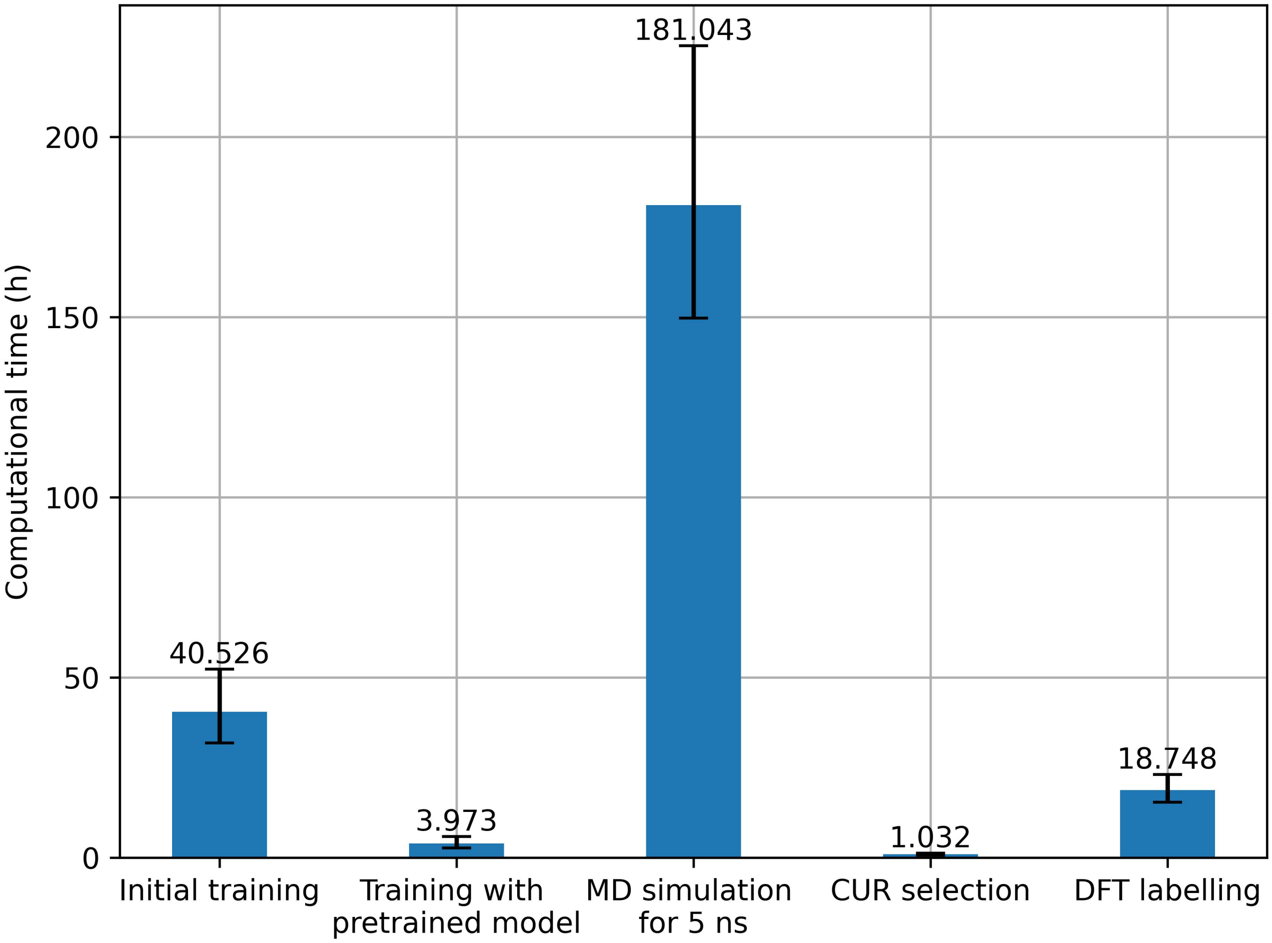

Figure S13: Computational time for training an initial model, retraining model, 5 ns NNPs MD simulation, and CUR selection. The training time of the initial model is evaluated on 1000,000 steps for five different models. By loading pretrained model parameters, retraining takes approximately 100,000 steps on average to early-stopping. DFT labelling cost is estimated by the time of labelling 100 structures using 40 CPU cores.

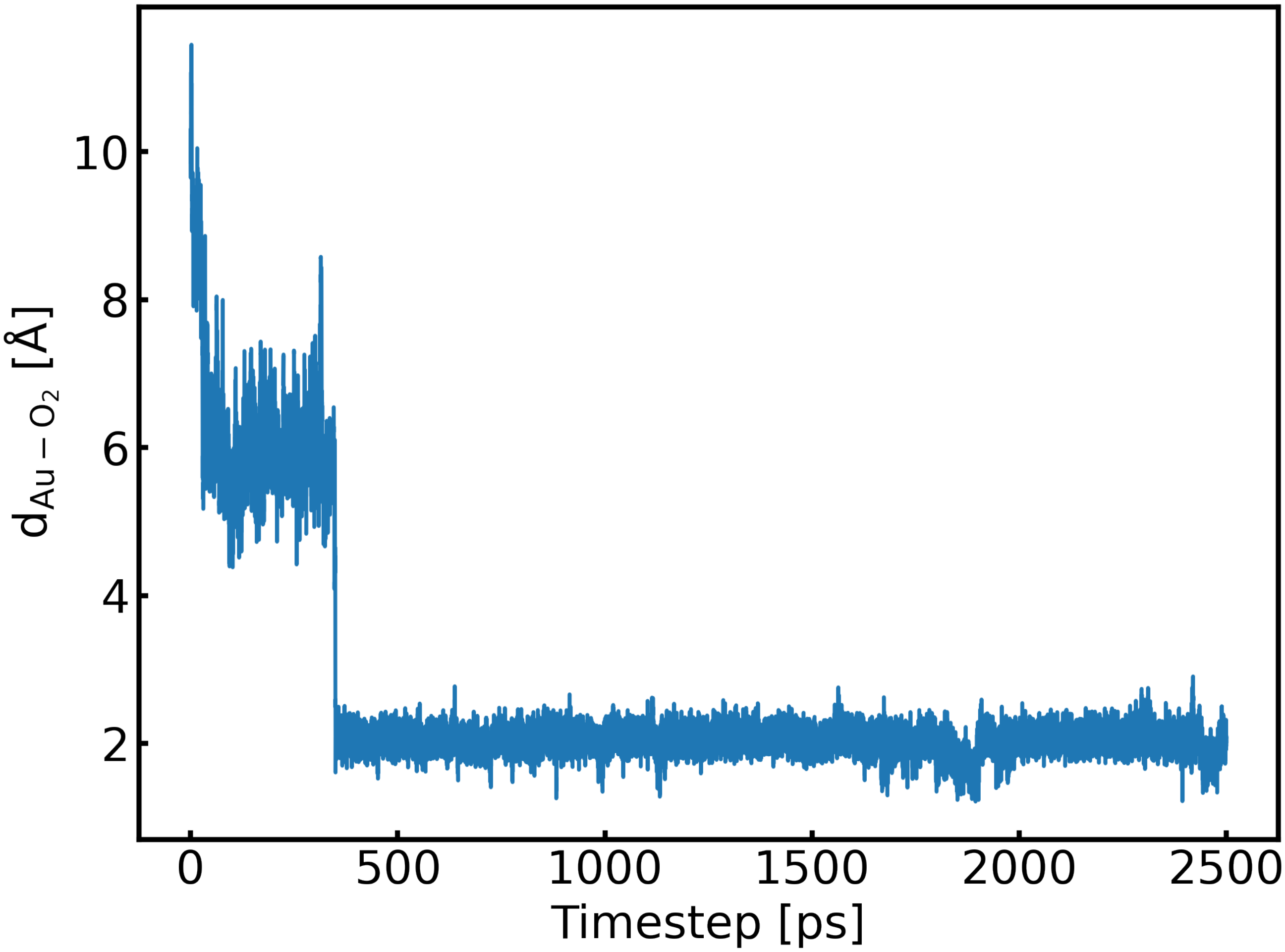

Figure S14: Evolution of the distance between O_2 and Au(100) surface along 2.5 ns MD simulation

References

[1] S. K. Natarajan and J. Behler, *Physical Chemistry Chemical Physics*, 2016, **18**, 28704–28725.

[2] V. Quaranta, M. Hellstrom and J. Behler, *The journal of physical chemistry letters*, 2017, **8**, 1476–1483.

[3] M. Yang, L. Bonati, D. Polino and M. Parrinello, *Catalysis Today*, 2022, **387**, 143–149.

[4] R. He, H. Wu, L. Zhang, X. Wang, F. Fu, S. Liu and Z. Zhong, *Physical Review B*, 2022, **105**, 064104.

[5] Y.-B. Liu, J.-Y. Yang, G.-M. Xin, L.-H. Liu, G. Csányi and B.-Y. Cao, *The Journal of Chemical Physics*, 2020, **153**, 144501.

[6] E. Davidson, T. Daff, G. Csanyi and M. Finnis, *Physical Review Materials*, 2020, **4**, 063804.

[7] W. Hu, M. Shuaibi, A. Das, S. Goyal, A. Sriram, J. Leskovec, D. Parikh and C. L. Zitnick, *arXiv preprint arXiv:2103.01436*, 2021.

[8] L. Chanussot, A. Das, S. Goyal, T. Lavril, M. Shuaibi, M. Riviere, K. Tran, J. Heras-Domingo, C. Ho, W. Hu *et al.*, *ACS Catalysis*, 2021, **11**, 6059–6072.

[9] J. Gasteiger, M. Shuaibi, A. Sriram, S. Günnemann, Z. Ulissi, C. L. Zitnick and A. Das, *arXiv preprint arXiv:2204.02782*, 2022.

[10] Z. Li, K. Meidani, P. Yadav and A. Barati Farimani, *The Journal of Chemical Physics*, 2022, **156**, 144103.

[11] S. Batzner, A. Musaelian, L. Sun, M. Geiger, J. P. Mailoa, M. Kornbluth, N. Molinari, T. E. Smidt and B. Kozinsky, *Nature Communications*, 2022, **13**, 1–11.
